# Supplementary figures and images for: Precision Identification of Locally Advanced Rectal Cancer in Denoised CT Scans Using EfficientNet and Voting System Algorithms
Source: Bioengineering (Basel). 2024 Apr 19;11(4):399. doi: 10.3390/bioengineering11040399 (PMC11048699; doi:10.3390/bioengineering11040399)

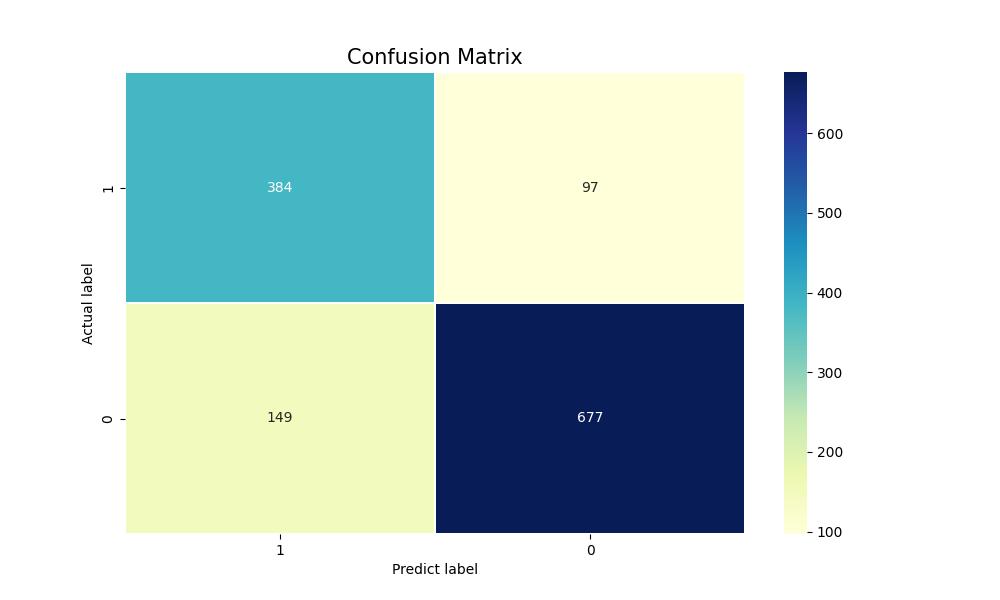

Supplement: Supplementary file 1 [file bioengineering-11-00399-s001.zip › Supplement 1/Model 1 CRM positive Images result/Model 1 Confusion Matrix Test 1 Image.jpg]

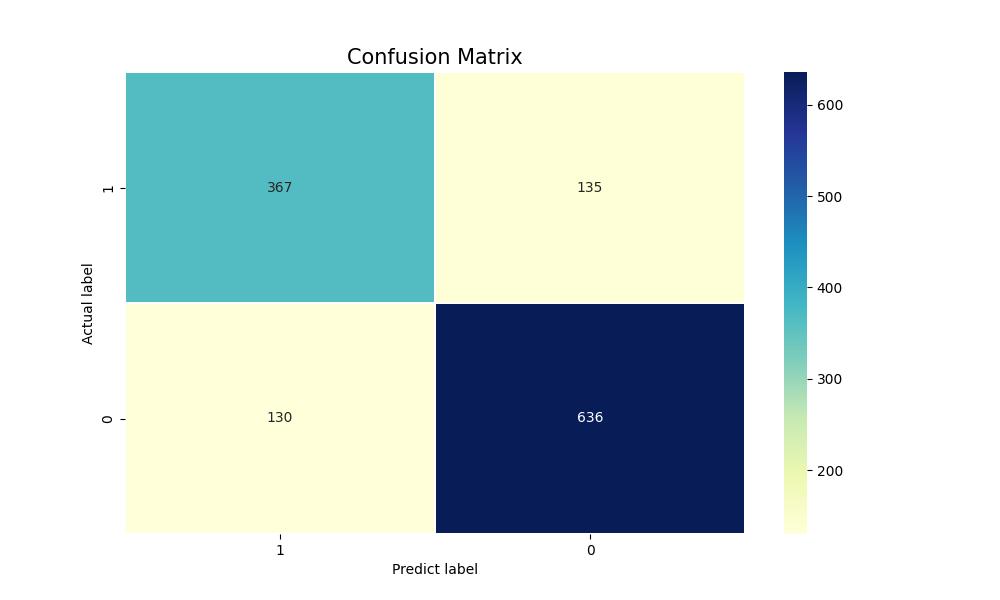

Supplement: Supplementary file 1 [file bioengineering-11-00399-s001.zip › Supplement 1/Model 1 CRM positive Images result/Model 1 Confusion Matrix Test 2 Image.jpg]

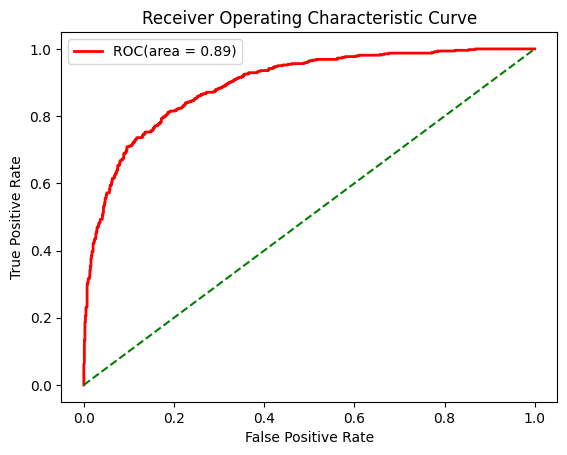

Supplement: Supplementary file 1 [file bioengineering-11-00399-s001.zip › Supplement 1/Model 1 CRM positive Images result/Model 1 ROC Test 1 Image.png]

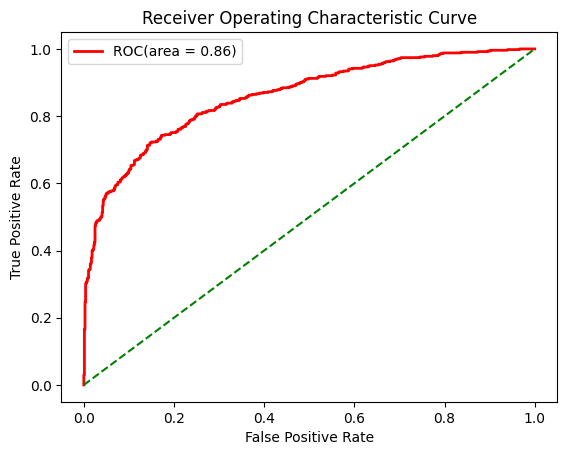

Supplement: Supplementary file 1 [file bioengineering-11-00399-s001.zip › Supplement 1/Model 1 CRM positive Images result/Model 1 ROC Test 2 Image.png]

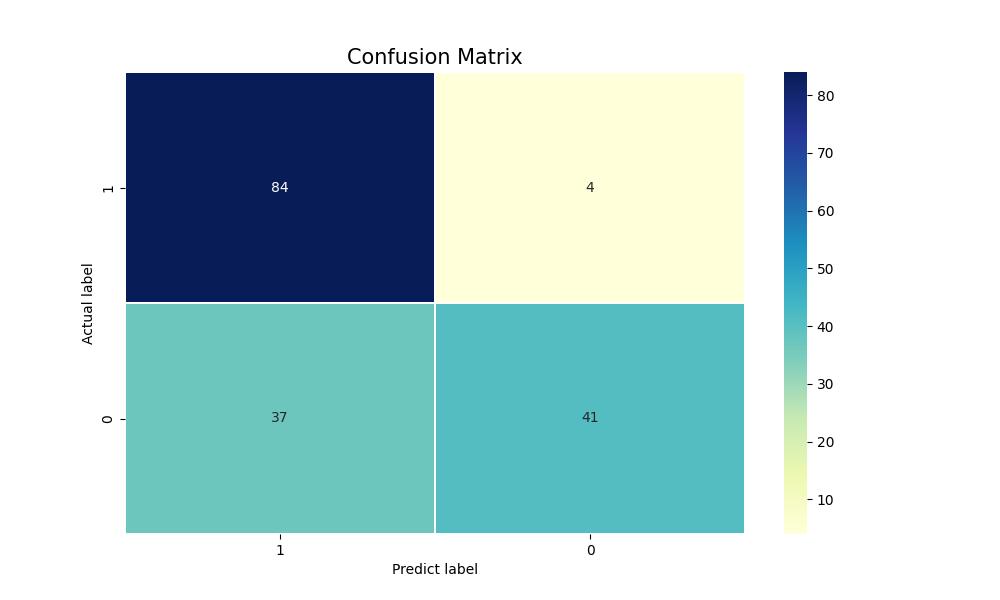

Supplement: Supplementary file 1 [file bioengineering-11-00399-s001.zip › Supplement 2/Model 1 LARC case result/Test 1/Hard vote LARC AUC Confu. Matrix/Model 1 Confusion Matrix T1 HardVote +any one.jpg]

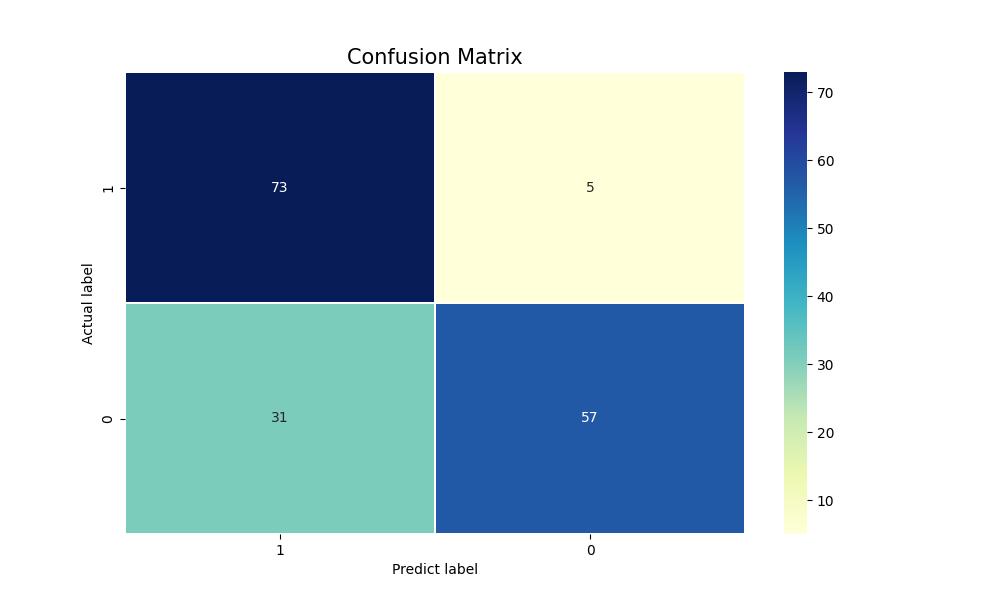

Supplement: Supplementary file 1 [file bioengineering-11-00399-s001.zip › Supplement 2/Model 1 LARC case result/Test 1/Hard vote LARC AUC Confu. Matrix/Model 1 Confusion Matrix T1 HardVote 0.20.jpg]

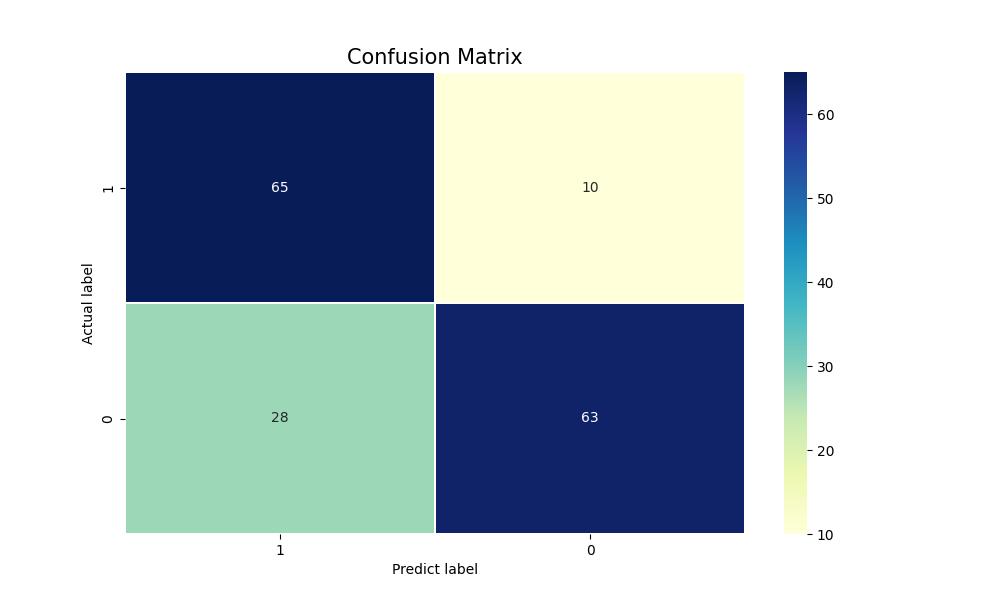

Supplement: Supplementary file 1 [file bioengineering-11-00399-s001.zip › Supplement 2/Model 1 LARC case result/Test 1/Hard vote LARC AUC Confu. Matrix/Model 1 Confusion Matrix T1 HardVote 0.25.jpg]

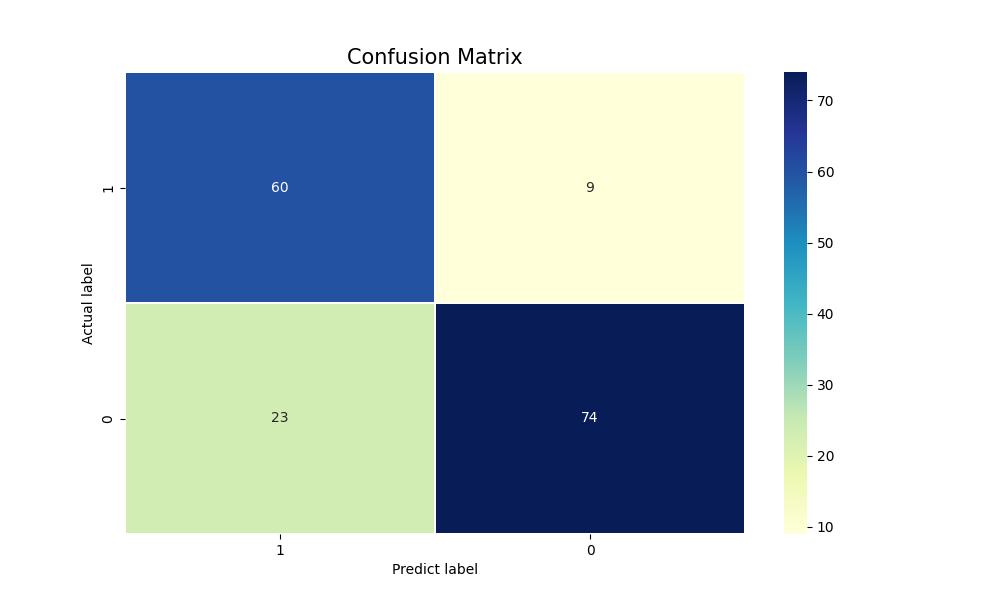

Supplement: Supplementary file 1 [file bioengineering-11-00399-s001.zip › Supplement 2/Model 1 LARC case result/Test 1/Hard vote LARC AUC Confu. Matrix/Model 1 Confusion Matrix T1 HardVote 0.30.jpg]

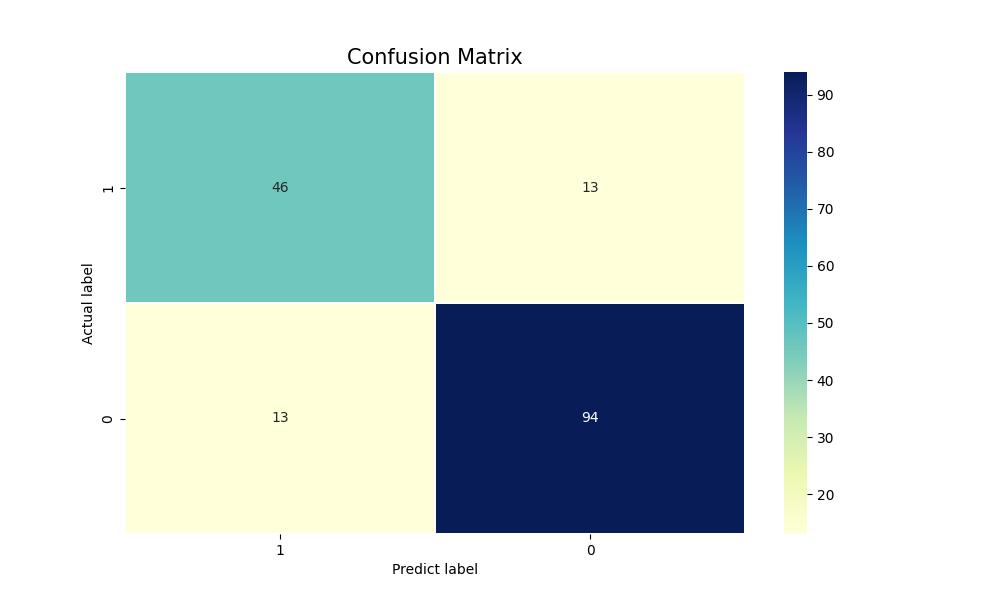

Supplement: Supplementary file 1 [file bioengineering-11-00399-s001.zip › Supplement 2/Model 1 LARC case result/Test 1/Hard vote LARC AUC Confu. Matrix/Model 1 Confusion Matrix T1 HardVote 0.50.jpg]

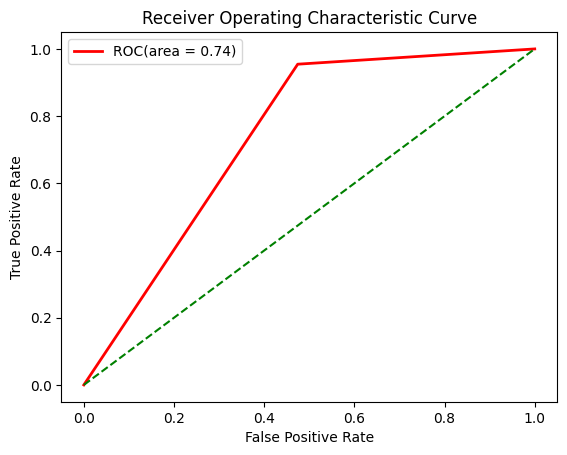

Supplement: Supplementary file 1 [file bioengineering-11-00399-s001.zip › Supplement 2/Model 1 LARC case result/Test 1/Hard vote LARC AUC Confu. Matrix/Model 1 ROC T1 HardVote +any one.png]

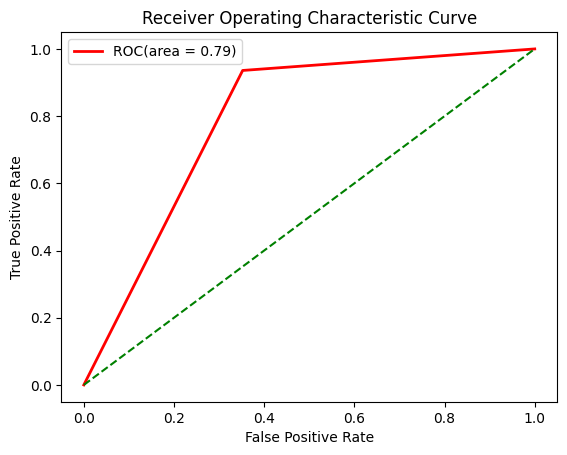

Supplement: Supplementary file 1 [file bioengineering-11-00399-s001.zip › Supplement 2/Model 1 LARC case result/Test 1/Hard vote LARC AUC Confu. Matrix/Model 1 ROC T1 HardVote 0.20.png]

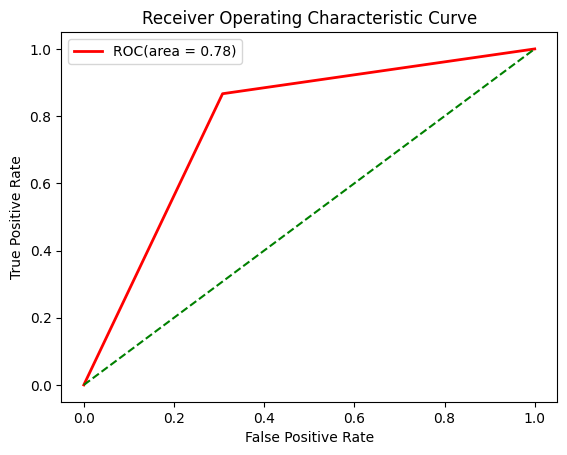

Supplement: Supplementary file 1 [file bioengineering-11-00399-s001.zip › Supplement 2/Model 1 LARC case result/Test 1/Hard vote LARC AUC Confu. Matrix/Model 1 ROC T1 HardVote 0.25.png]

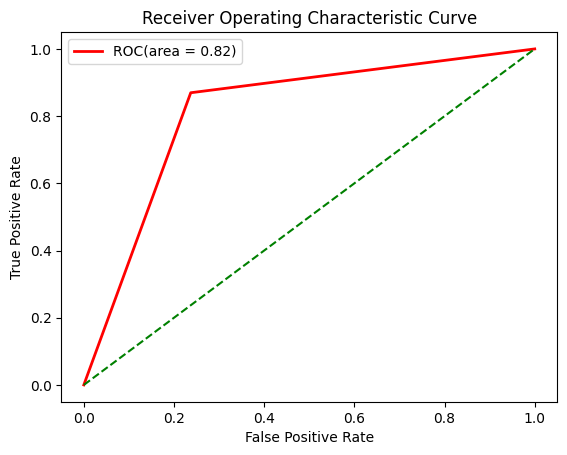

Supplement: Supplementary file 1 [file bioengineering-11-00399-s001.zip › Supplement 2/Model 1 LARC case result/Test 1/Hard vote LARC AUC Confu. Matrix/Model 1 ROC T1 HardVote 0.30.png]

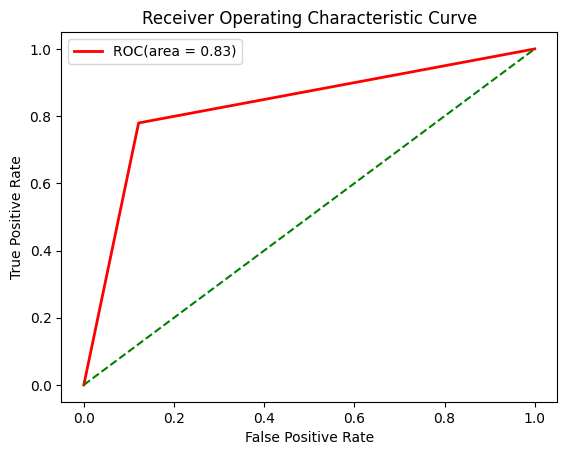

Supplement: Supplementary file 1 [file bioengineering-11-00399-s001.zip › Supplement 2/Model 1 LARC case result/Test 1/Hard vote LARC AUC Confu. Matrix/Model 1 ROC T1 HardVote 0.50.png]

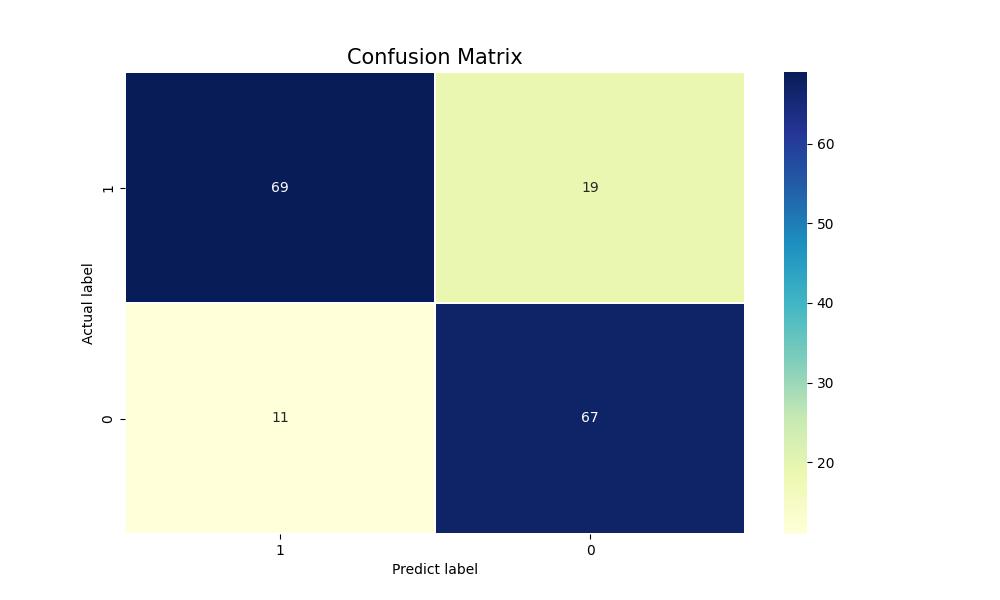

Supplement: Supplementary file 1 [file bioengineering-11-00399-s001.zip › Supplement 2/Model 1 LARC case result/Test 1/Soft vote LARC AUC Confu. Matrix/Model 1 Confusion Matrix T1 SoftVote +any one.jpg]

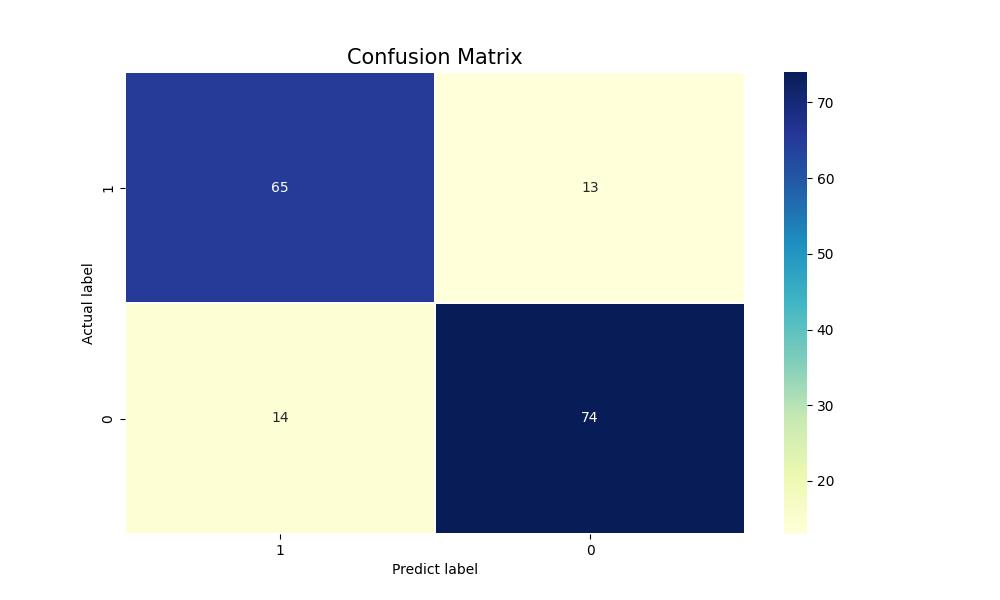

Supplement: Supplementary file 1 [file bioengineering-11-00399-s001.zip › Supplement 2/Model 1 LARC case result/Test 1/Soft vote LARC AUC Confu. Matrix/Model 1 Confusion Matrix T1 SoftVote 0.20.jpg]

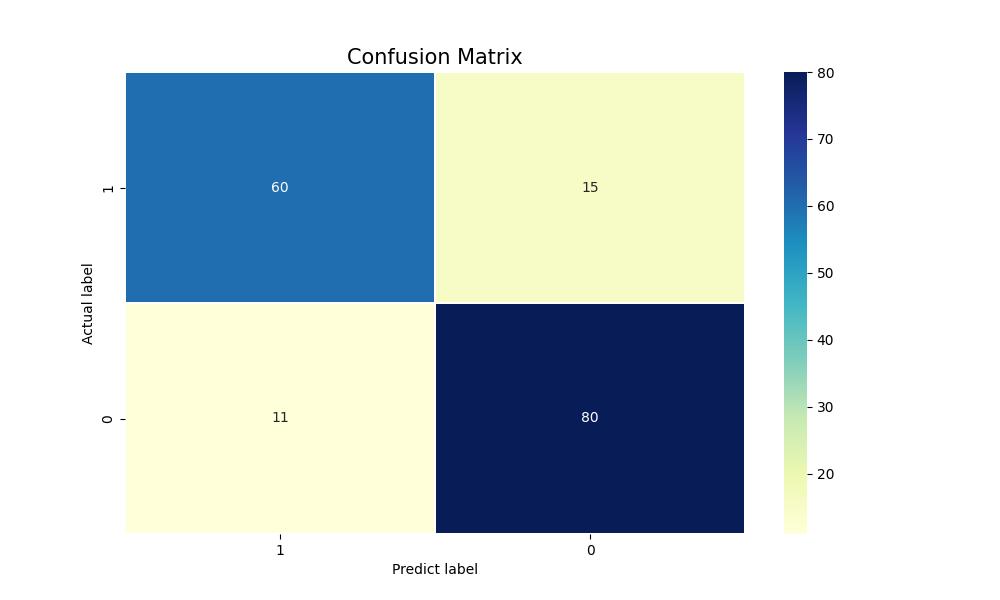

Supplement: Supplementary file 1 [file bioengineering-11-00399-s001.zip › Supplement 2/Model 1 LARC case result/Test 1/Soft vote LARC AUC Confu. Matrix/Model 1 Confusion Matrix T1 SoftVote 0.25.jpg]

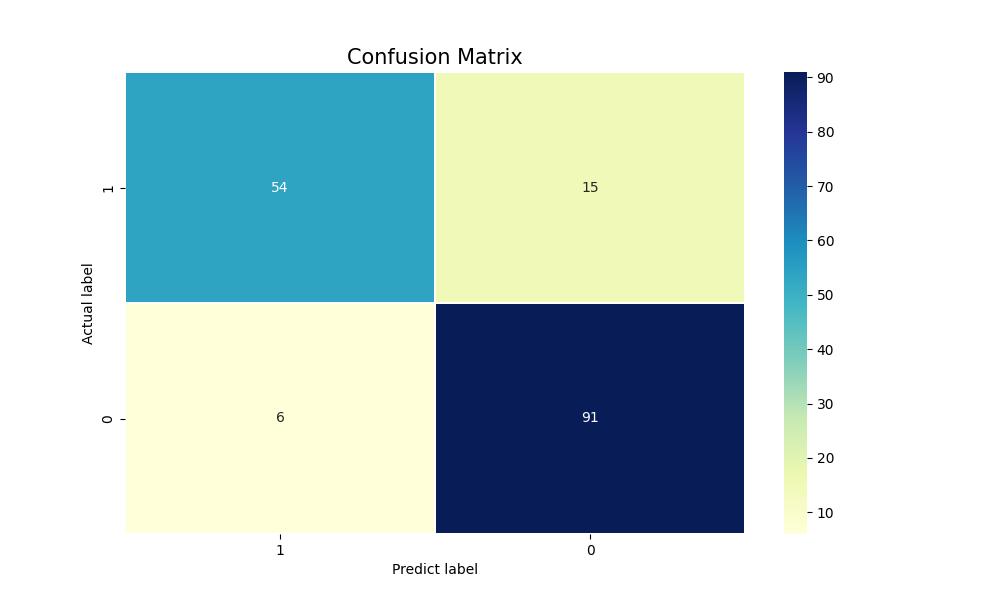

Supplement: Supplementary file 1 [file bioengineering-11-00399-s001.zip › Supplement 2/Model 1 LARC case result/Test 1/Soft vote LARC AUC Confu. Matrix/Model 1 Confusion Matrix T1 SoftVote 0.33.jpg]

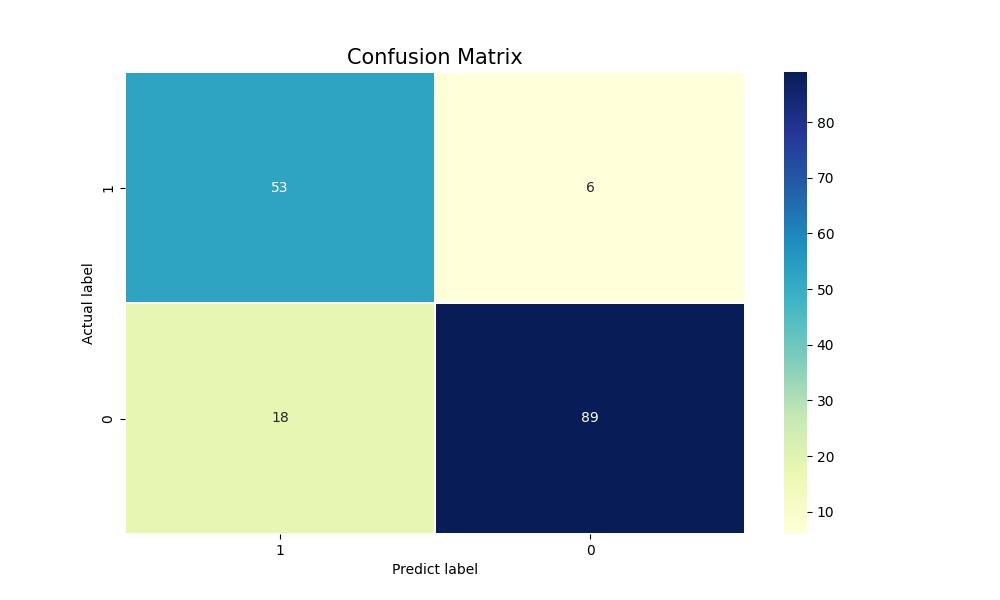

Supplement: Supplementary file 1 [file bioengineering-11-00399-s001.zip › Supplement 2/Model 1 LARC case result/Test 1/Soft vote LARC AUC Confu. Matrix/Model 1 Confusion Matrix T1 SoftVote 0.50.jpg]

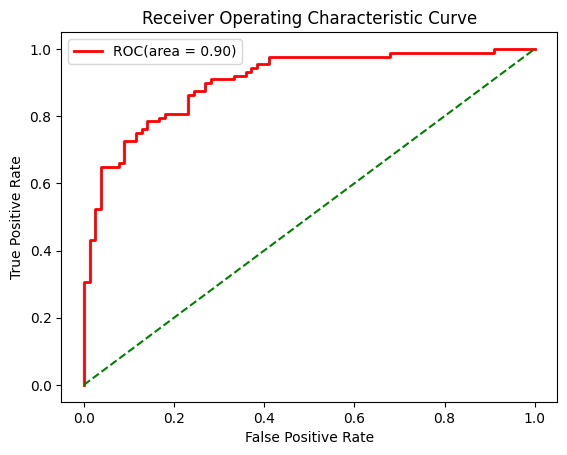

Supplement: Supplementary file 1 [file bioengineering-11-00399-s001.zip › Supplement 2/Model 1 LARC case result/Test 1/Soft vote LARC AUC Confu. Matrix/Model 1 ROC T1 SoftVote +any one.png]

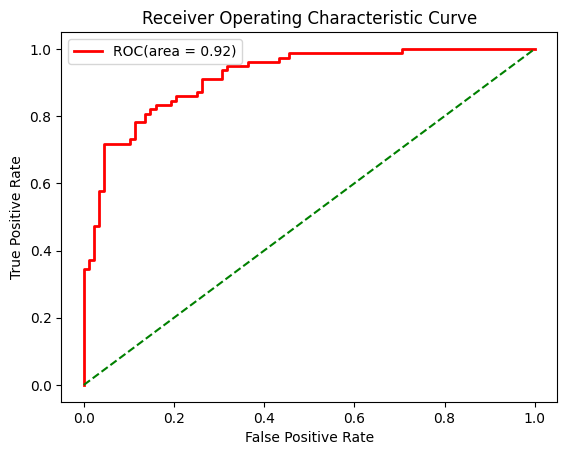

Supplement: Supplementary file 1 [file bioengineering-11-00399-s001.zip › Supplement 2/Model 1 LARC case result/Test 1/Soft vote LARC AUC Confu. Matrix/Model 1 ROC T1 SoftVote 0.20.png]

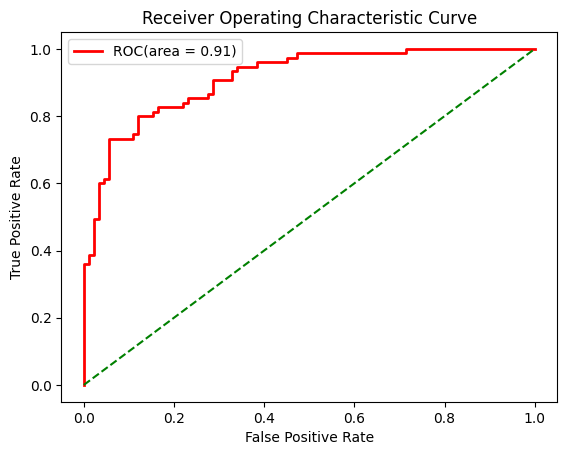

Supplement: Supplementary file 1 [file bioengineering-11-00399-s001.zip › Supplement 2/Model 1 LARC case result/Test 1/Soft vote LARC AUC Confu. Matrix/Model 1 ROC T1 SoftVote 0.25.png]

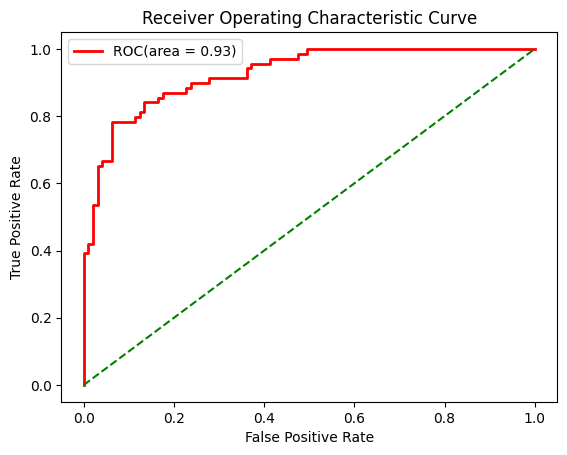

Supplement: Supplementary file 1 [file bioengineering-11-00399-s001.zip › Supplement 2/Model 1 LARC case result/Test 1/Soft vote LARC AUC Confu. Matrix/Model 1 ROC T1 SoftVote 0.33.png]

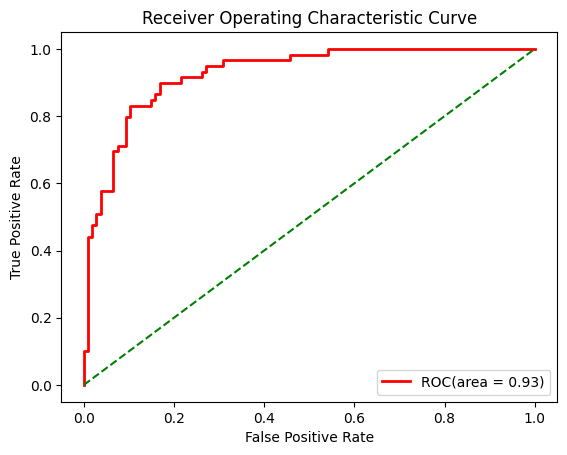

Supplement: Supplementary file 1 [file bioengineering-11-00399-s001.zip › Supplement 2/Model 1 LARC case result/Test 1/Soft vote LARC AUC Confu. Matrix/Model 1 ROC T1 SoftVote 0.50.png]

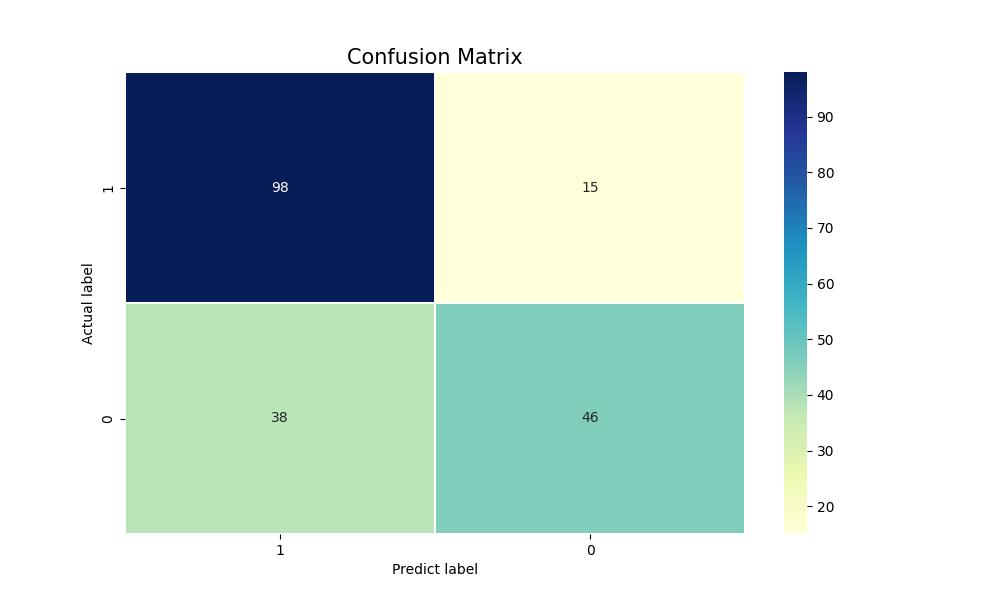

Supplement: Supplementary file 1 [file bioengineering-11-00399-s001.zip › Supplement 2/Model 1 LARC case result/Test 2/Hard vote LARC AUC Confu. Matrix/Model 1 Conf Matr T2 HardVote +any one.jpg]

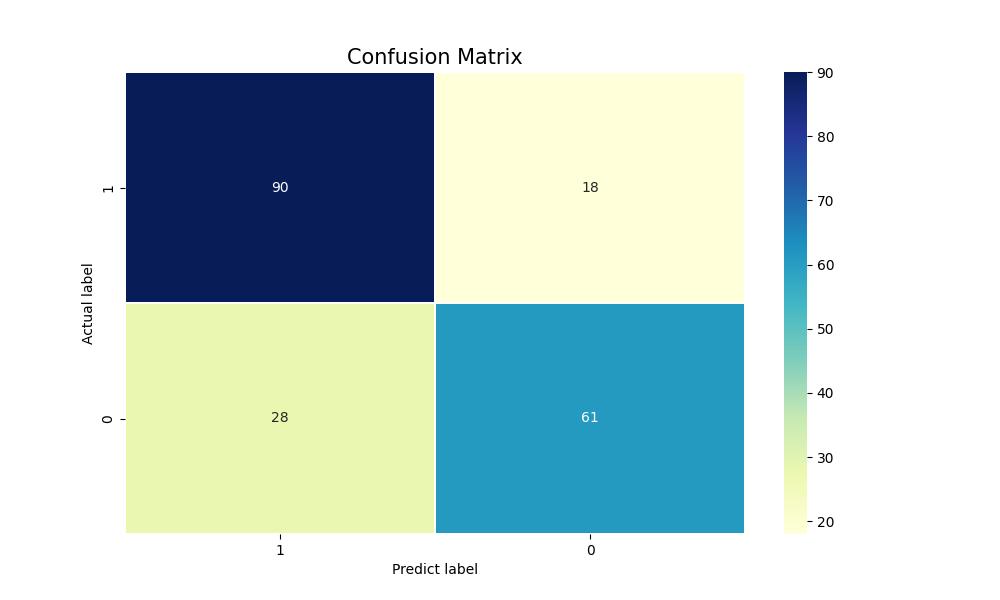

Supplement: Supplementary file 1 [file bioengineering-11-00399-s001.zip › Supplement 2/Model 1 LARC case result/Test 2/Hard vote LARC AUC Confu. Matrix/Model 1 Conf Matr T2 HardVote 0.20.jpg]

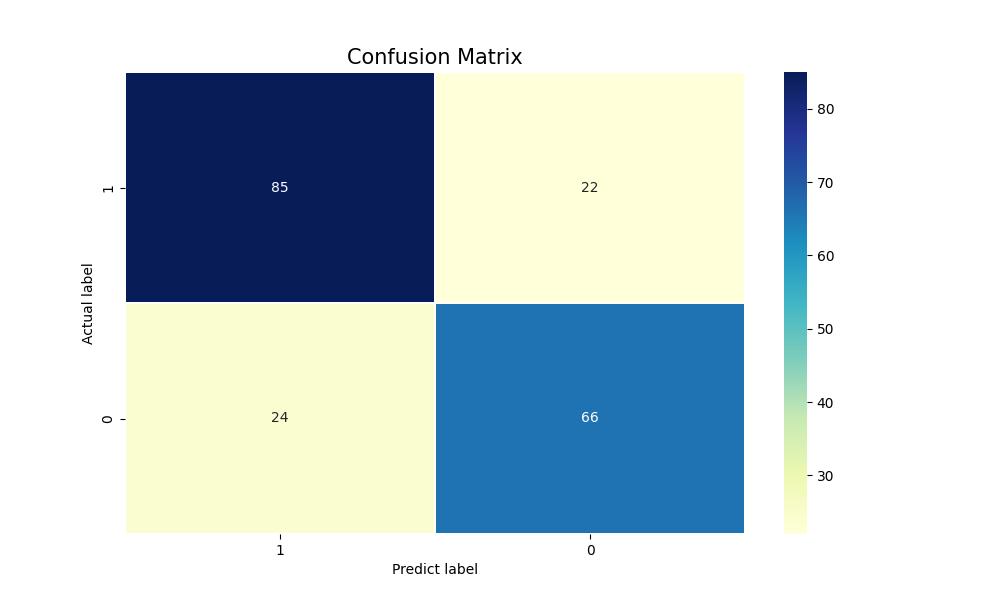

Supplement: Supplementary file 1 [file bioengineering-11-00399-s001.zip › Supplement 2/Model 1 LARC case result/Test 2/Hard vote LARC AUC Confu. Matrix/Model 1 Conf Matr T2 HardVote 0.25.jpg]

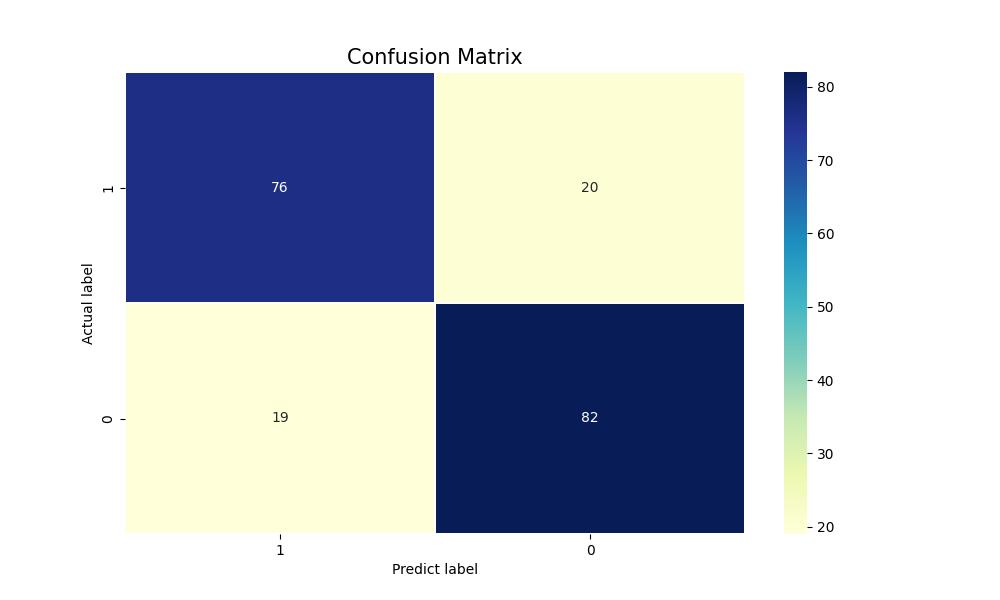

Supplement: Supplementary file 1 [file bioengineering-11-00399-s001.zip › Supplement 2/Model 1 LARC case result/Test 2/Hard vote LARC AUC Confu. Matrix/Model 1 Conf Matr T2 HardVote 0.33.jpg]

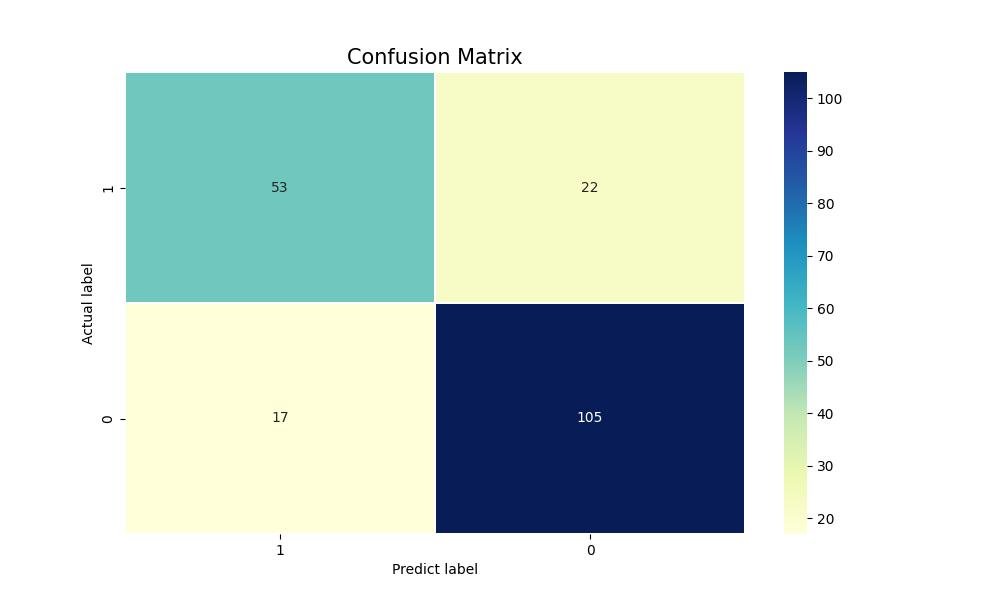

Supplement: Supplementary file 1 [file bioengineering-11-00399-s001.zip › Supplement 2/Model 1 LARC case result/Test 2/Hard vote LARC AUC Confu. Matrix/Model 1 Conf Matr T2 HardVote 0.50.jpg]

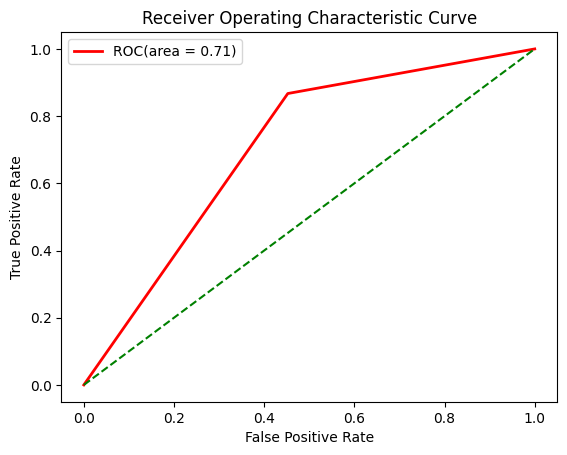

Supplement: Supplementary file 1 [file bioengineering-11-00399-s001.zip › Supplement 2/Model 1 LARC case result/Test 2/Hard vote LARC AUC Confu. Matrix/Model 1 ROC T2 HardVote +any one.png]

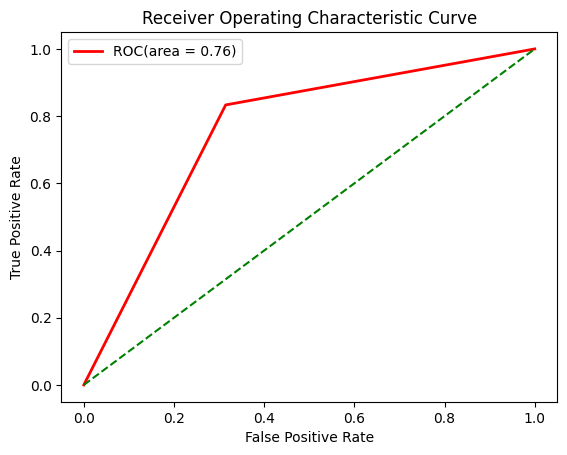

Supplement: Supplementary file 1 [file bioengineering-11-00399-s001.zip › Supplement 2/Model 1 LARC case result/Test 2/Hard vote LARC AUC Confu. Matrix/Model 1 ROC T2 HardVote 0.20.png]

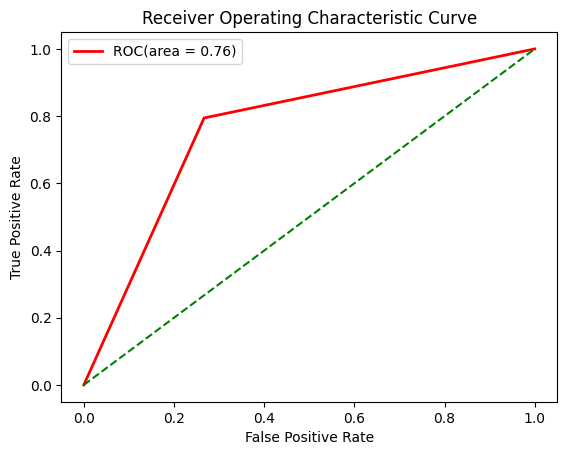

Supplement: Supplementary file 1 [file bioengineering-11-00399-s001.zip › Supplement 2/Model 1 LARC case result/Test 2/Hard vote LARC AUC Confu. Matrix/Model 1 ROC T2 HardVote 0.25.png]

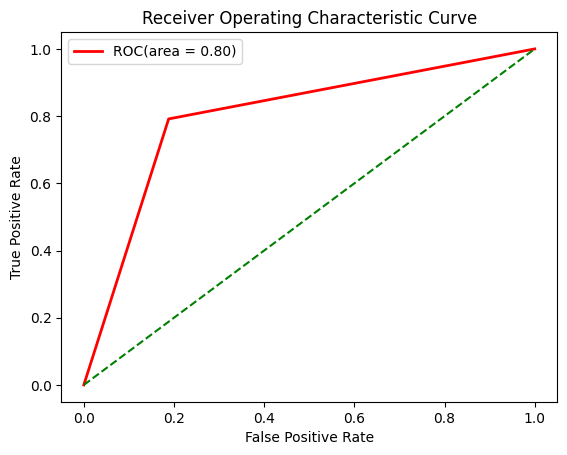

Supplement: Supplementary file 1 [file bioengineering-11-00399-s001.zip › Supplement 2/Model 1 LARC case result/Test 2/Hard vote LARC AUC Confu. Matrix/Model 1 ROC T2 HardVote 0.33.png]

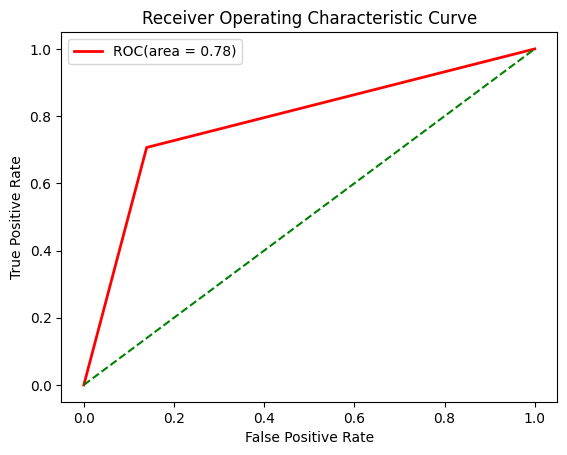

Supplement: Supplementary file 1 [file bioengineering-11-00399-s001.zip › Supplement 2/Model 1 LARC case result/Test 2/Hard vote LARC AUC Confu. Matrix/Model 1 ROC T2 HardVote 0.50.png]

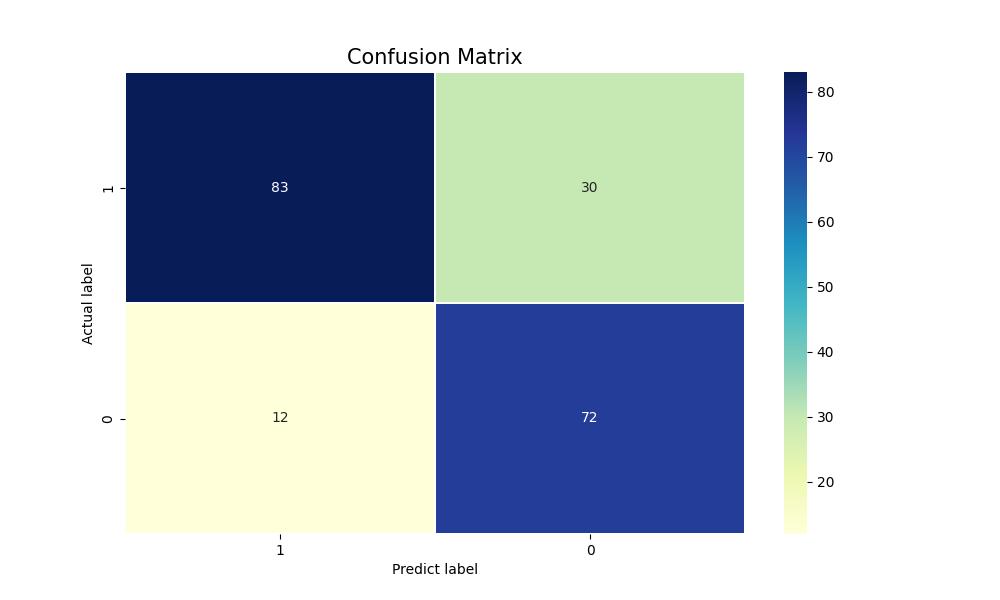

Supplement: Supplementary file 1 [file bioengineering-11-00399-s001.zip › Supplement 2/Model 1 LARC case result/Test 2/Soft vote LARC AUC Confu. Matrix/Model 1 Conf Matr T2 SoftVote +any one.jpg]

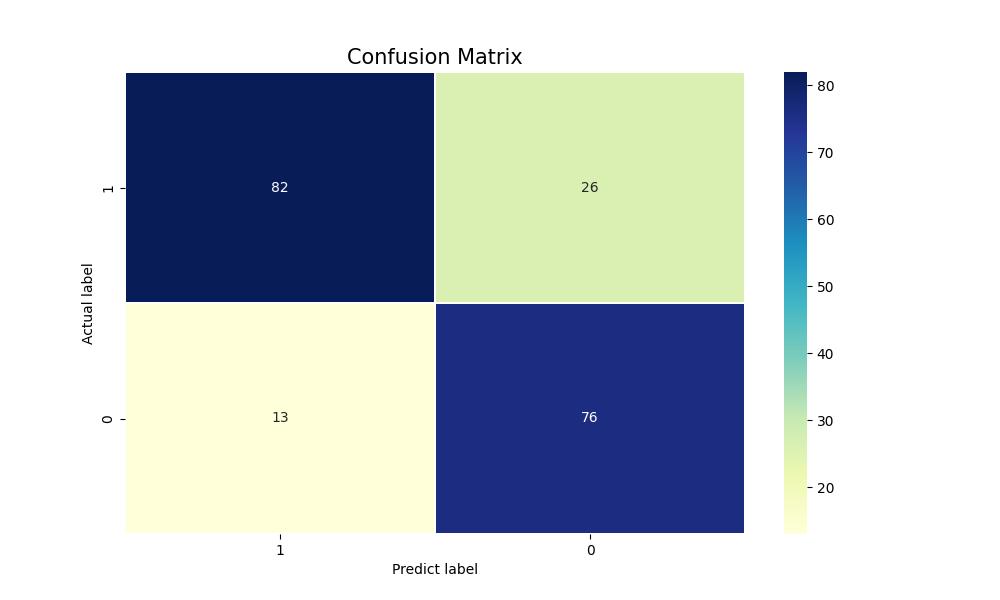

Supplement: Supplementary file 1 [file bioengineering-11-00399-s001.zip › Supplement 2/Model 1 LARC case result/Test 2/Soft vote LARC AUC Confu. Matrix/Model 1 Conf Matr T2 SoftVote 0.20.jpg]

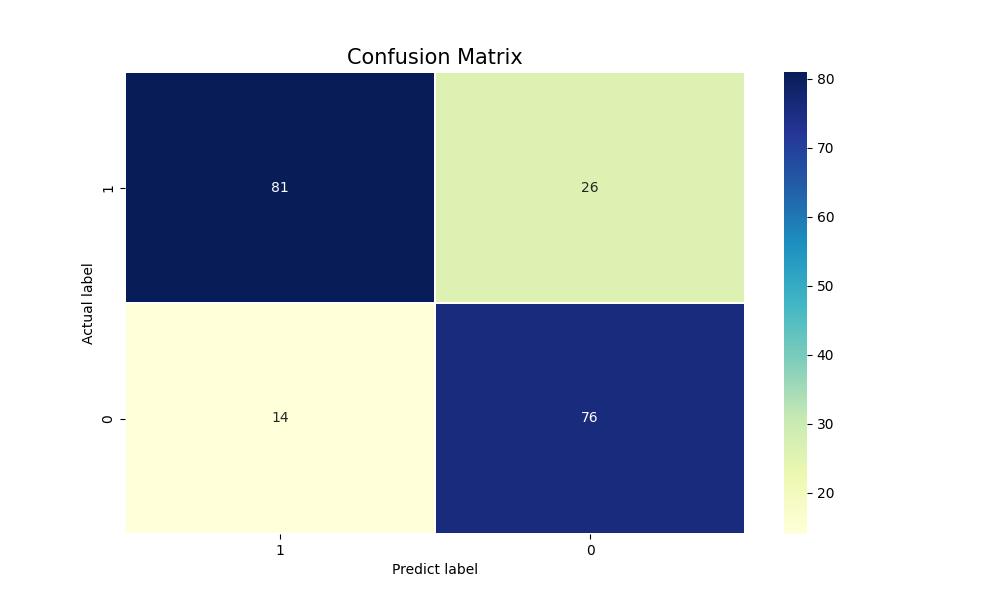

Supplement: Supplementary file 1 [file bioengineering-11-00399-s001.zip › Supplement 2/Model 1 LARC case result/Test 2/Soft vote LARC AUC Confu. Matrix/Model 1 Conf Matr T2 SoftVote 0.25.jpg]

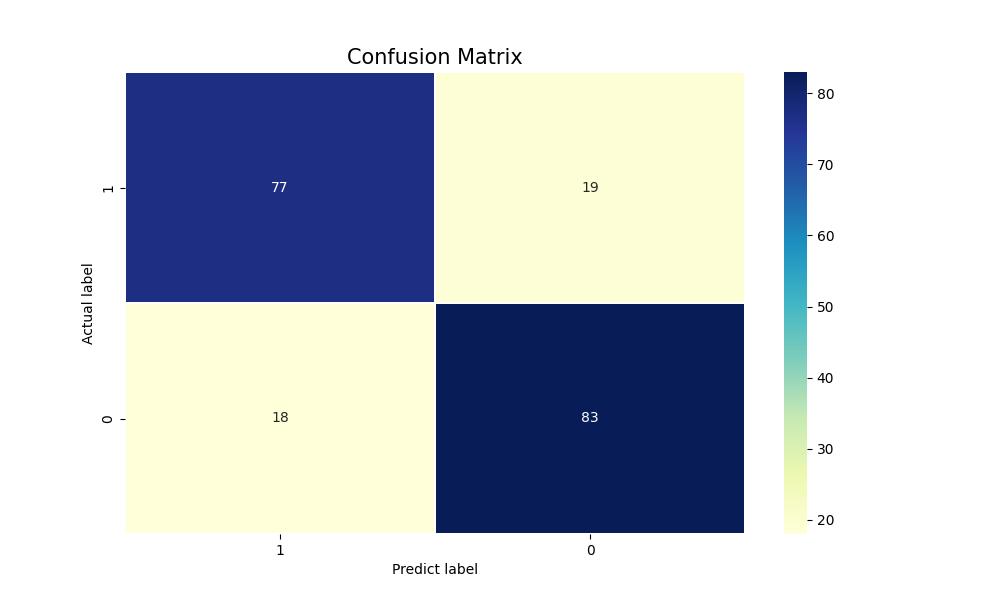

Supplement: Supplementary file 1 [file bioengineering-11-00399-s001.zip › Supplement 2/Model 1 LARC case result/Test 2/Soft vote LARC AUC Confu. Matrix/Model 1 Conf Matr T2 SoftVote 0.33.jpg]

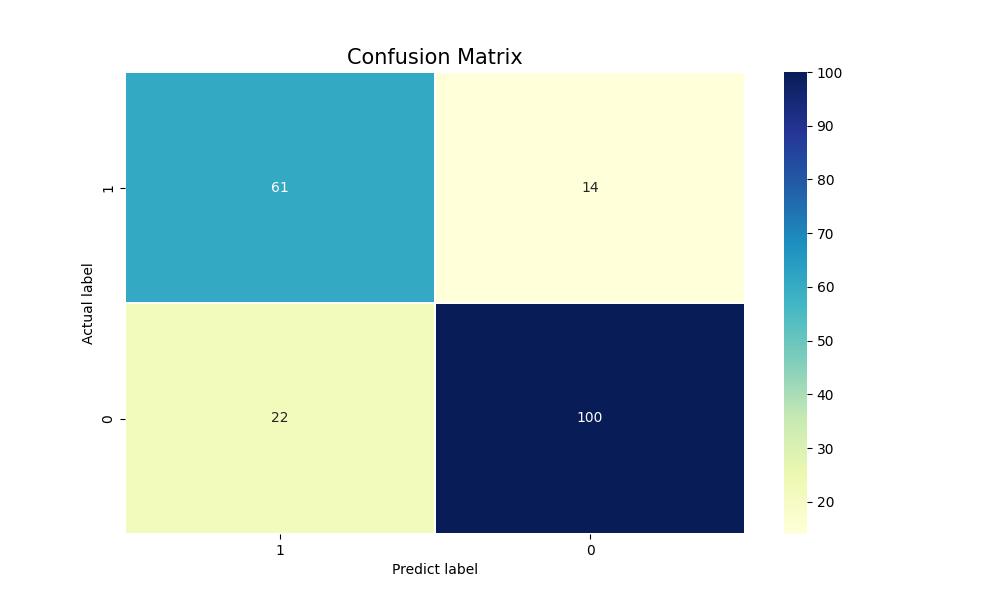

Supplement: Supplementary file 1 [file bioengineering-11-00399-s001.zip › Supplement 2/Model 1 LARC case result/Test 2/Soft vote LARC AUC Confu. Matrix/Model 1 Conf Matr T2 SoftVote 0.50.jpg]

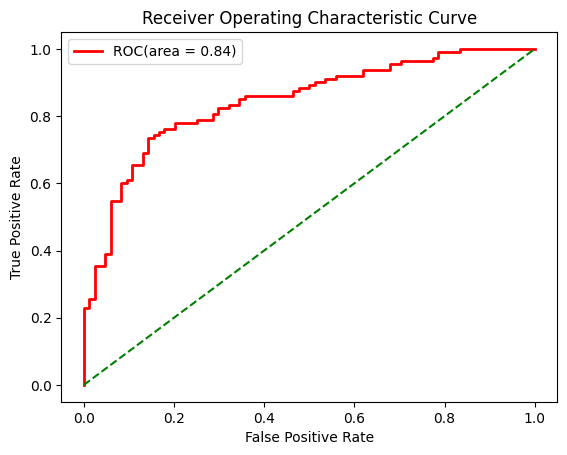

Supplement: Supplementary file 1 [file bioengineering-11-00399-s001.zip › Supplement 2/Model 1 LARC case result/Test 2/Soft vote LARC AUC Confu. Matrix/Model 1 ROC T2 SoftVote +any one.png]

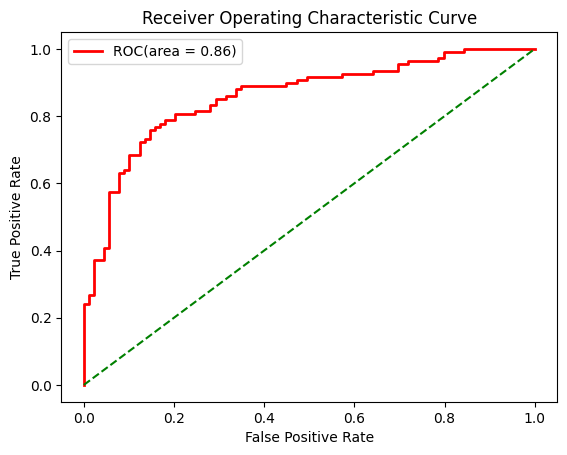

Supplement: Supplementary file 1 [file bioengineering-11-00399-s001.zip › Supplement 2/Model 1 LARC case result/Test 2/Soft vote LARC AUC Confu. Matrix/Model 1 ROC T2 SoftVote 0.20.png]

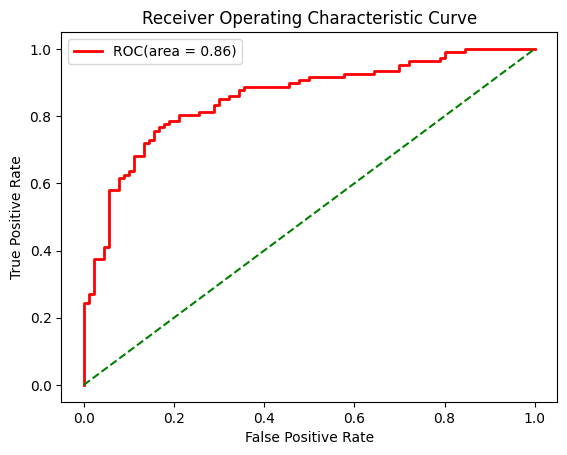

Supplement: Supplementary file 1 [file bioengineering-11-00399-s001.zip › Supplement 2/Model 1 LARC case result/Test 2/Soft vote LARC AUC Confu. Matrix/Model 1 ROC T2 SoftVote 0.25.png]

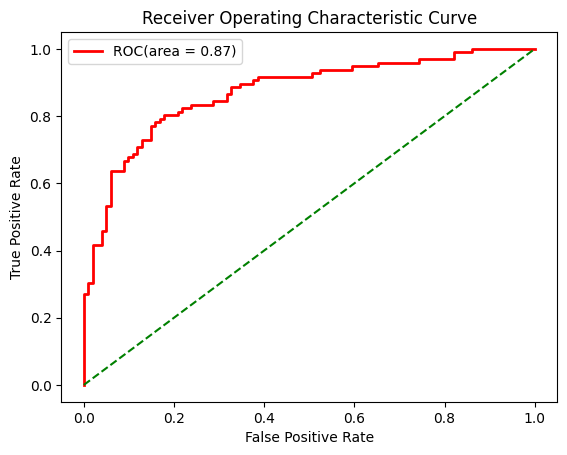

Supplement: Supplementary file 1 [file bioengineering-11-00399-s001.zip › Supplement 2/Model 1 LARC case result/Test 2/Soft vote LARC AUC Confu. Matrix/Model 1 ROC T2 SoftVote 0.33.png]

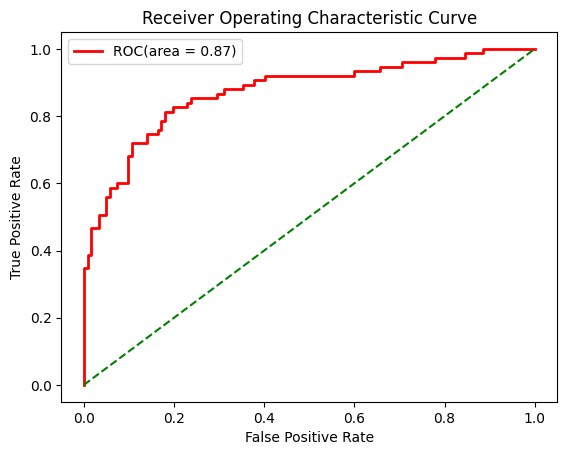

Supplement: Supplementary file 1 [file bioengineering-11-00399-s001.zip › Supplement 2/Model 1 LARC case result/Test 2/Soft vote LARC AUC Confu. Matrix/Model 1 ROC T2 SoftVote 0.50.png]

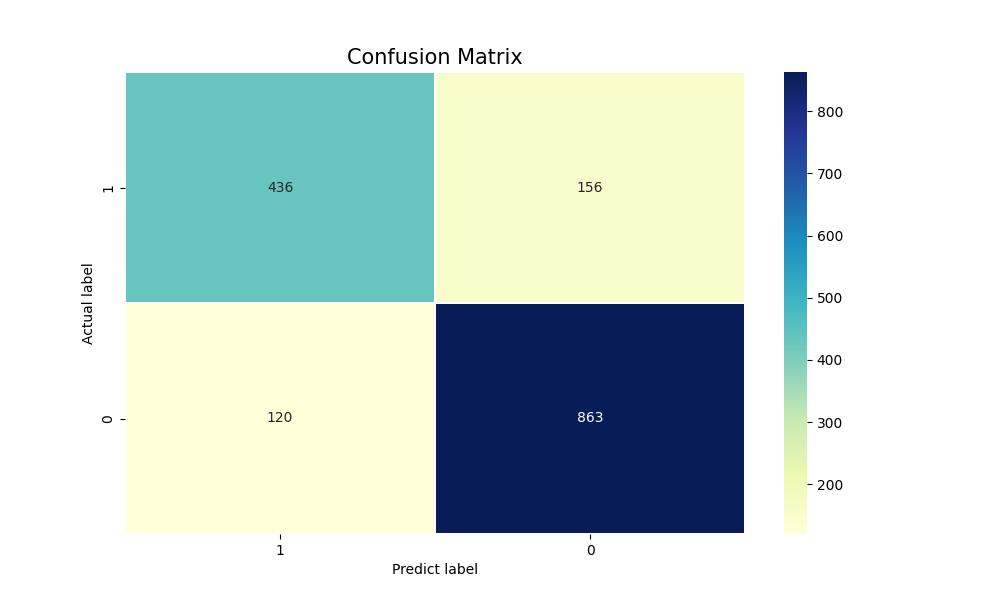

Supplement: Supplementary file 1 [file bioengineering-11-00399-s001.zip › Supplement 3/Model 2a 2b LARC case result/Model 2a Test 1 set 137 cases/CRM positive Images AUC Confu. matrix/Model 2a Confusion Matrix Test 1 Image.jpg]

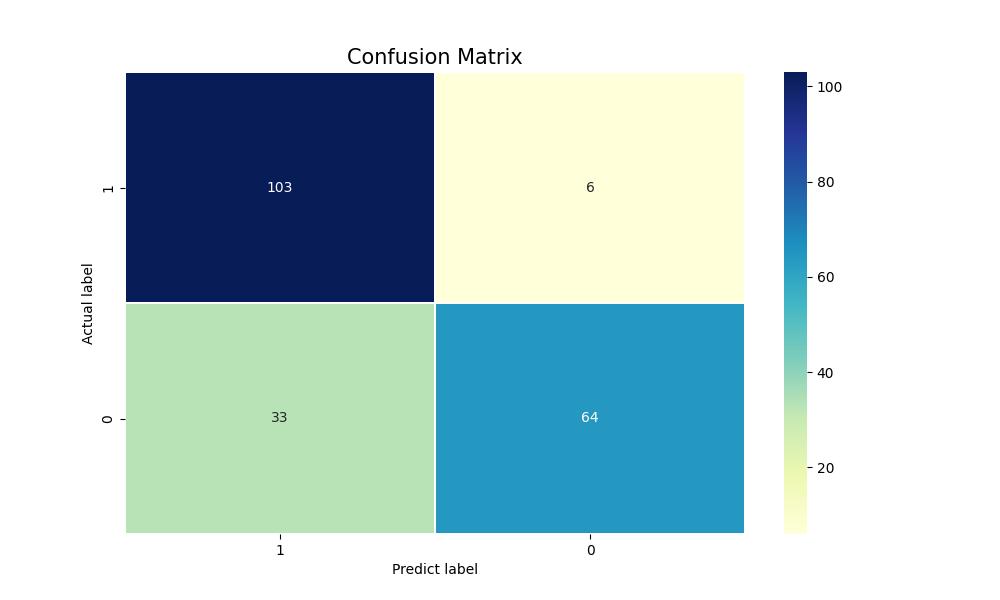

Supplement: Supplementary file 1 [file bioengineering-11-00399-s001.zip › Supplement 3/Model 2a 2b LARC case result/Model 2a Test 1 set 137 cases/Hard vote LARC AUC Confu. Matrix/Model 2a Confusion Matrix T1 HardVote +any one.jpg]

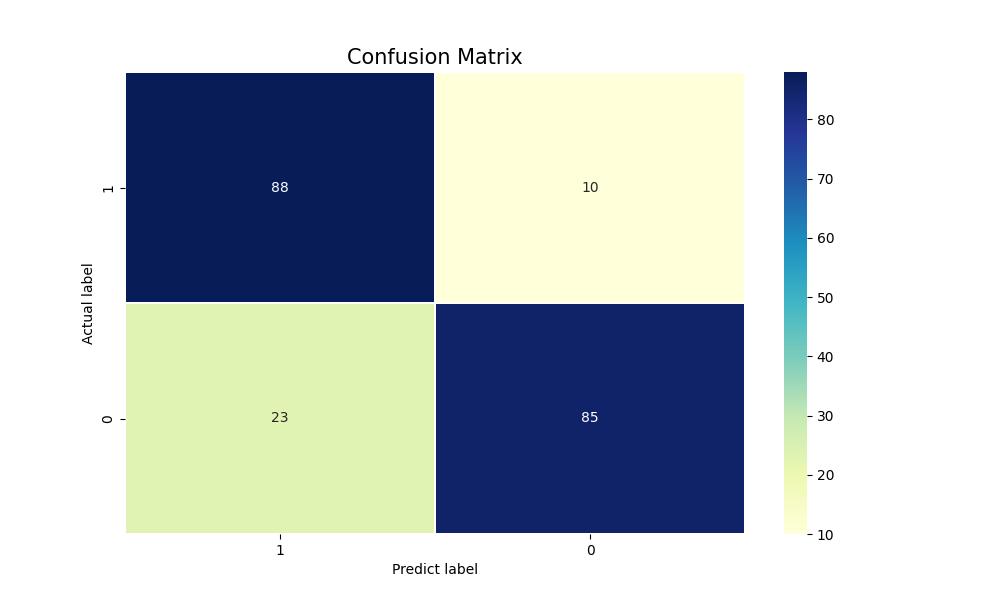

Supplement: Supplementary file 1 [file bioengineering-11-00399-s001.zip › Supplement 3/Model 2a 2b LARC case result/Model 2a Test 1 set 137 cases/Hard vote LARC AUC Confu. Matrix/Model 2a Confusion Matrix T1 HardVote 0.20.jpg]

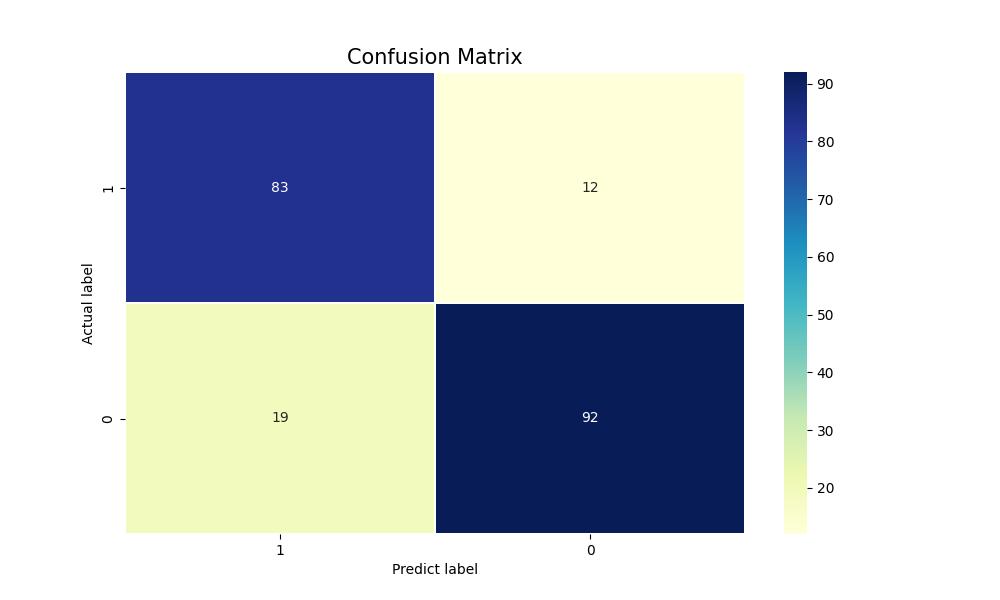

Supplement: Supplementary file 1 [file bioengineering-11-00399-s001.zip › Supplement 3/Model 2a 2b LARC case result/Model 2a Test 1 set 137 cases/Hard vote LARC AUC Confu. Matrix/Model 2a Confusion Matrix T1 HardVote 0.25.jpg]

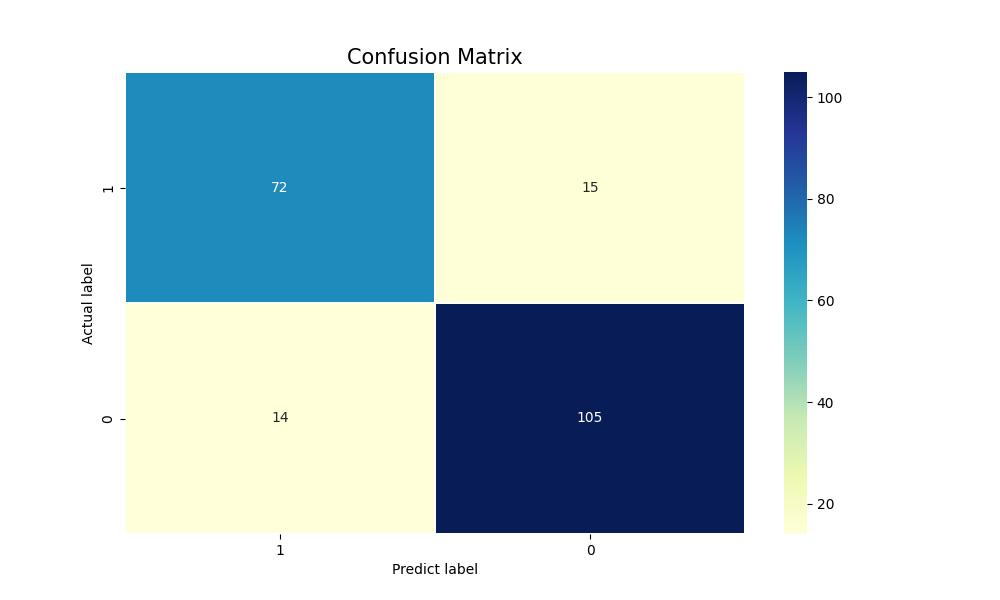

Supplement: Supplementary file 1 [file bioengineering-11-00399-s001.zip › Supplement 3/Model 2a 2b LARC case result/Model 2a Test 1 set 137 cases/Hard vote LARC AUC Confu. Matrix/Model 2a Confusion Matrix T1 HardVote 0.33.jpg]

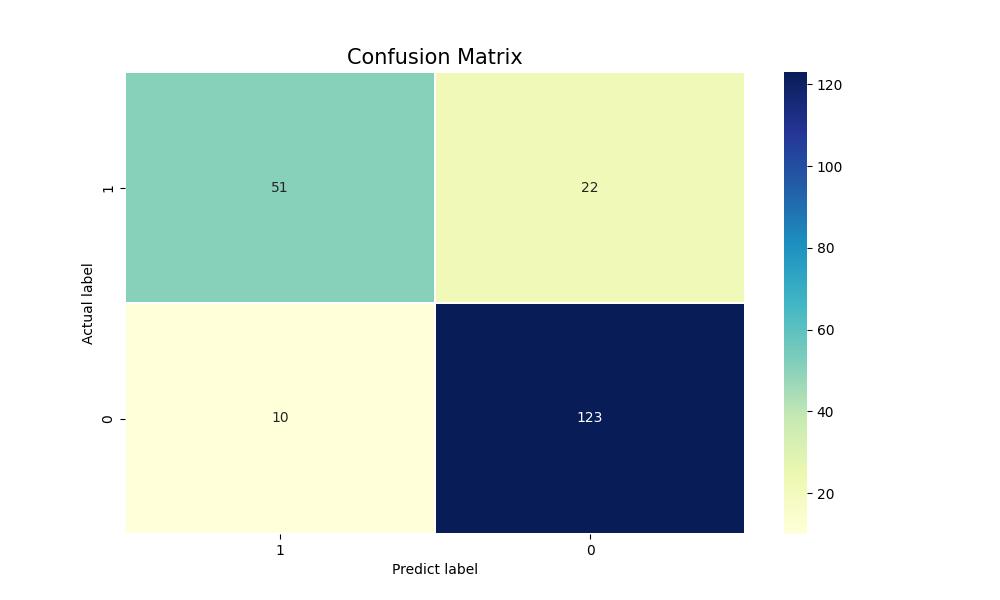

Supplement: Supplementary file 1 [file bioengineering-11-00399-s001.zip › Supplement 3/Model 2a 2b LARC case result/Model 2a Test 1 set 137 cases/Hard vote LARC AUC Confu. Matrix/Model 2a Confusion Matrix T1 HardVote 0.50.jpg]

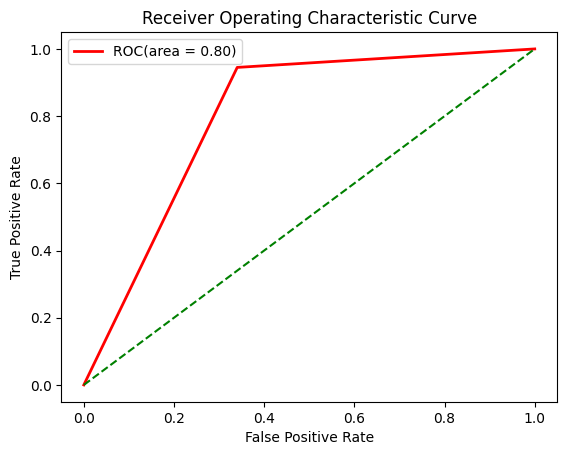

Supplement: Supplementary file 1 [file bioengineering-11-00399-s001.zip › Supplement 3/Model 2a 2b LARC case result/Model 2a Test 1 set 137 cases/Hard vote LARC AUC Confu. Matrix/Model 2a ROC T1 HardVote +any one.png]

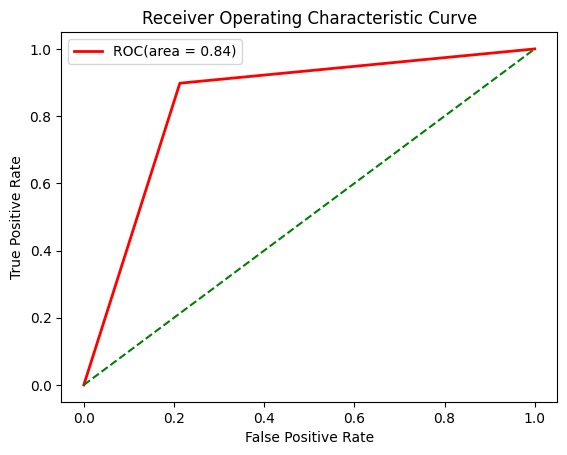

Supplement: Supplementary file 1 [file bioengineering-11-00399-s001.zip › Supplement 3/Model 2a 2b LARC case result/Model 2a Test 1 set 137 cases/Hard vote LARC AUC Confu. Matrix/Model 2a ROC T1 HardVote 0.20.png]

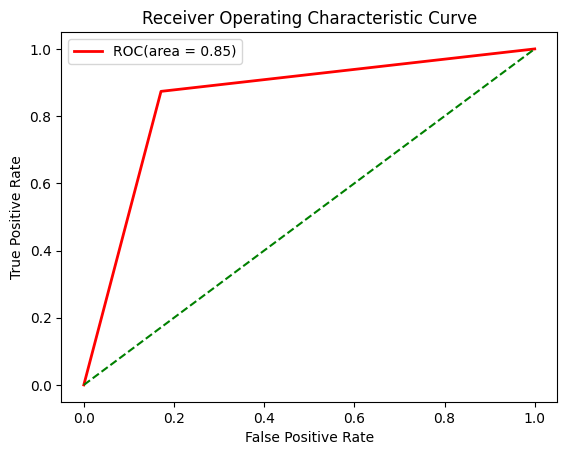

Supplement: Supplementary file 1 [file bioengineering-11-00399-s001.zip › Supplement 3/Model 2a 2b LARC case result/Model 2a Test 1 set 137 cases/Hard vote LARC AUC Confu. Matrix/Model 2a ROC T1 HardVote 0.25.png]

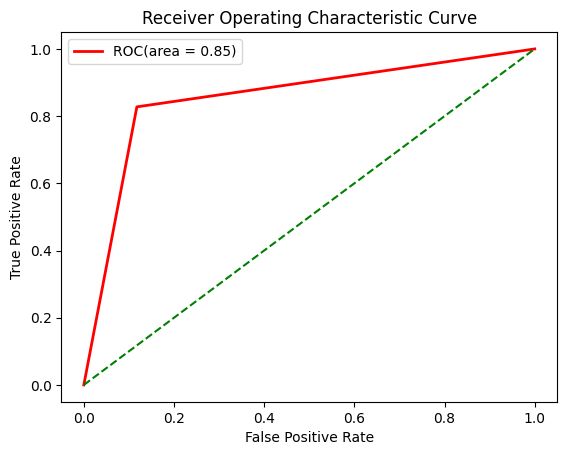

Supplement: Supplementary file 1 [file bioengineering-11-00399-s001.zip › Supplement 3/Model 2a 2b LARC case result/Model 2a Test 1 set 137 cases/Hard vote LARC AUC Confu. Matrix/Model 2a ROC T1 HardVote 0.33.png]

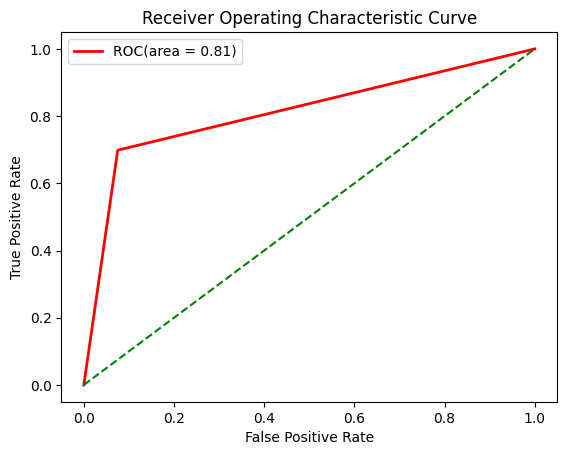

Supplement: Supplementary file 1 [file bioengineering-11-00399-s001.zip › Supplement 3/Model 2a 2b LARC case result/Model 2a Test 1 set 137 cases/Hard vote LARC AUC Confu. Matrix/Model 2a ROC T1 HardVote 0.50.png]

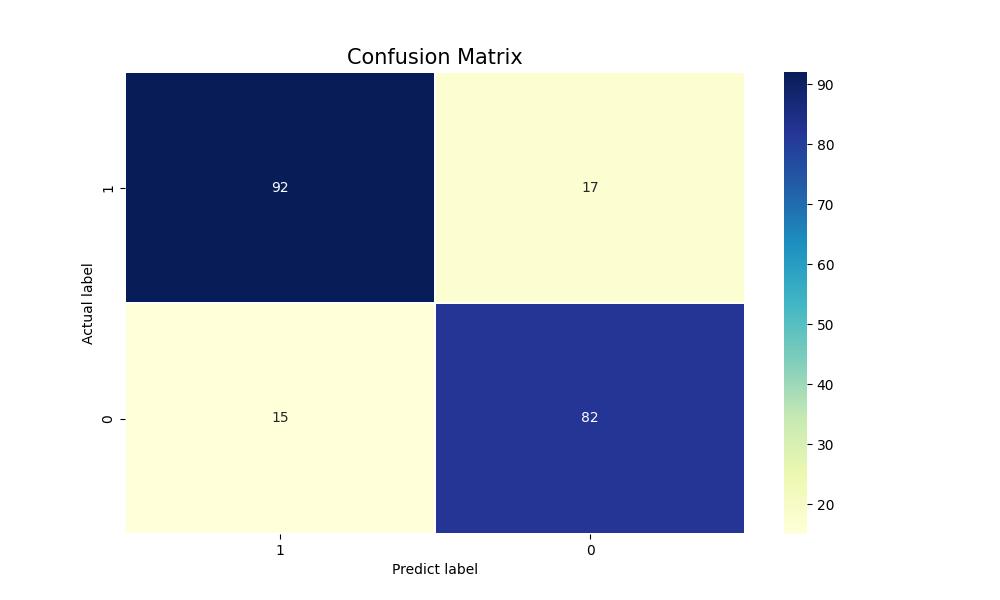

Supplement: Supplementary file 1 [file bioengineering-11-00399-s001.zip › Supplement 3/Model 2a 2b LARC case result/Model 2a Test 1 set 137 cases/Soft vote LARC AUC Confu. Matrix/Model 2a Confusion Matrix T1 SoftVote +any one.jpg]

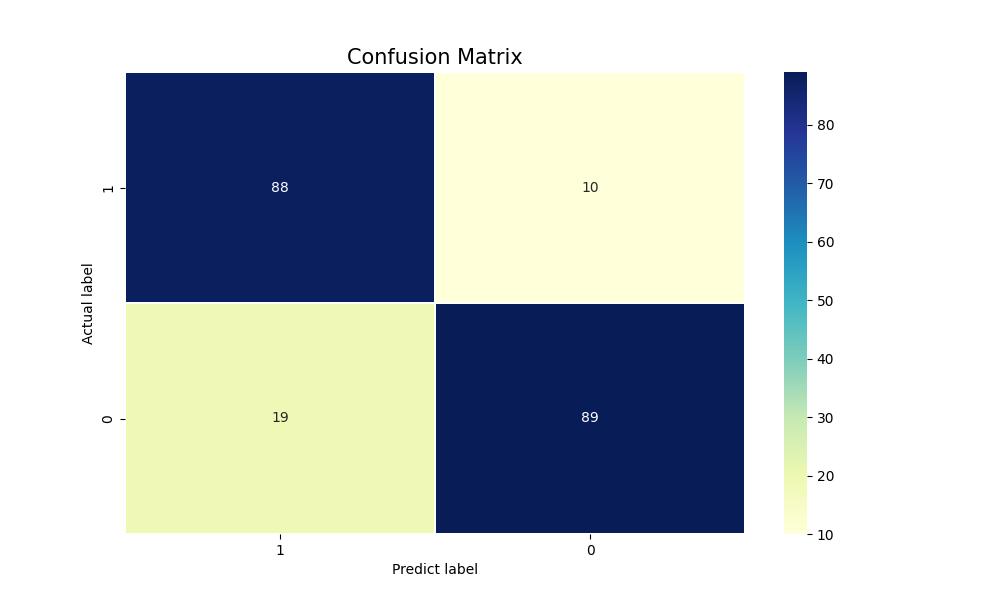

Supplement: Supplementary file 1 [file bioengineering-11-00399-s001.zip › Supplement 3/Model 2a 2b LARC case result/Model 2a Test 1 set 137 cases/Soft vote LARC AUC Confu. Matrix/Model 2a Confusion Matrix T1 SoftVote 0.20.jpg]

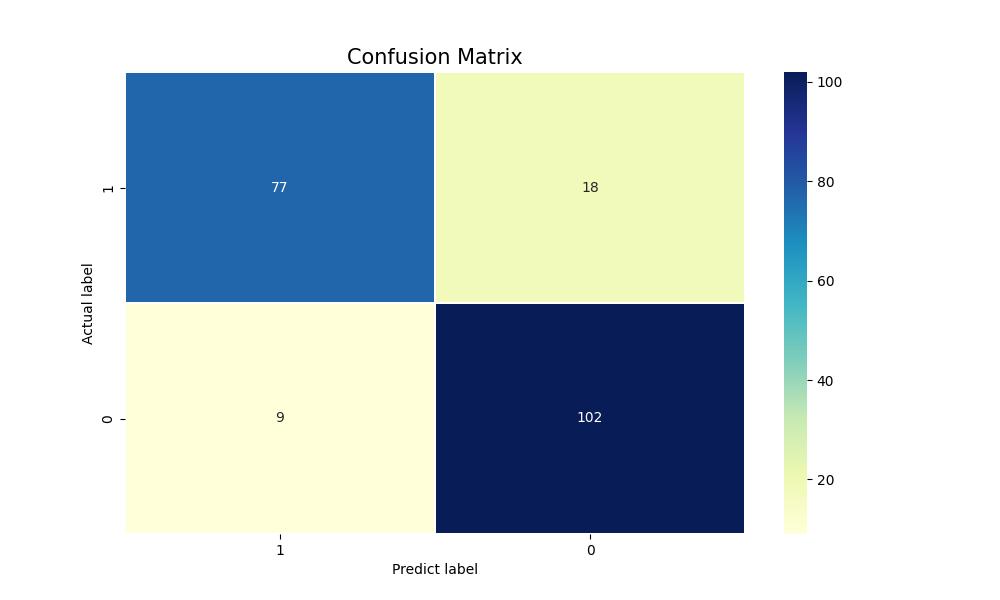

Supplement: Supplementary file 1 [file bioengineering-11-00399-s001.zip › Supplement 3/Model 2a 2b LARC case result/Model 2a Test 1 set 137 cases/Soft vote LARC AUC Confu. Matrix/Model 2a Confusion Matrix T1 SoftVote 0.25.jpg]

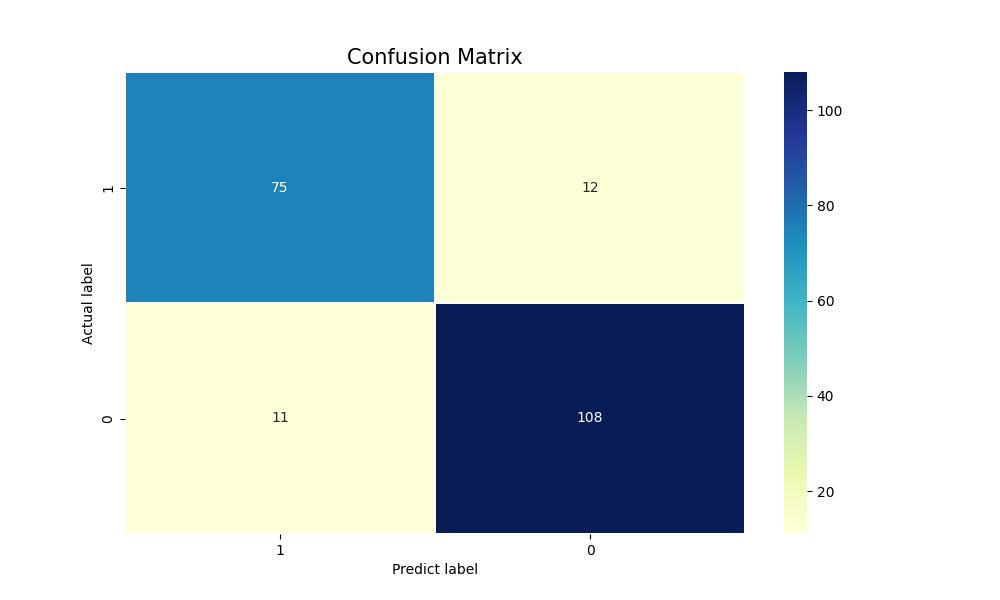

Supplement: Supplementary file 1 [file bioengineering-11-00399-s001.zip › Supplement 3/Model 2a 2b LARC case result/Model 2a Test 1 set 137 cases/Soft vote LARC AUC Confu. Matrix/Model 2a Confusion Matrix T1 SoftVote 0.33.jpg]

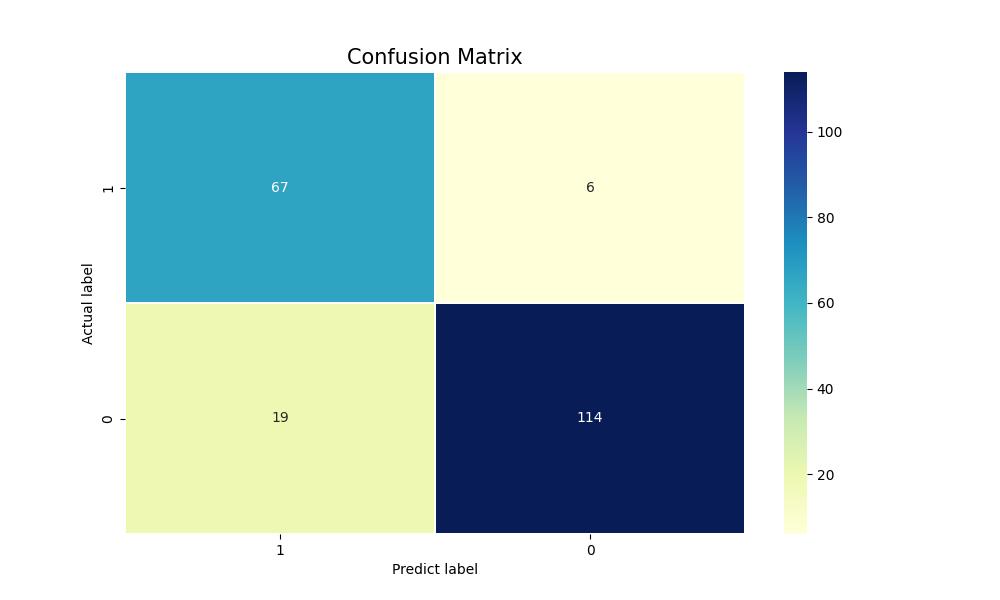

Supplement: Supplementary file 1 [file bioengineering-11-00399-s001.zip › Supplement 3/Model 2a 2b LARC case result/Model 2a Test 1 set 137 cases/Soft vote LARC AUC Confu. Matrix/Model 2a Confusion Matrix T1 SoftVote 0.50.jpg]

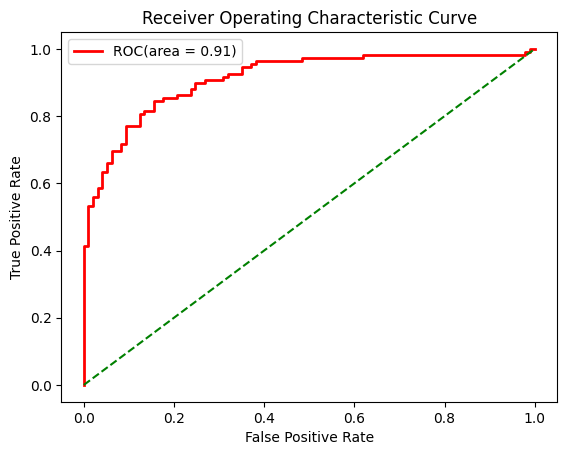

Supplement: Supplementary file 1 [file bioengineering-11-00399-s001.zip › Supplement 3/Model 2a 2b LARC case result/Model 2a Test 1 set 137 cases/Soft vote LARC AUC Confu. Matrix/Model 2a ROC T1 SoftVote +any one.png]

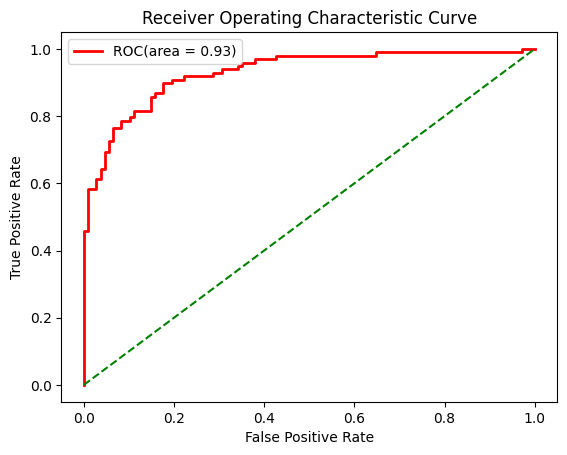

Supplement: Supplementary file 1 [file bioengineering-11-00399-s001.zip › Supplement 3/Model 2a 2b LARC case result/Model 2a Test 1 set 137 cases/Soft vote LARC AUC Confu. Matrix/Model 2a ROC T1 SoftVote 0.20.png]

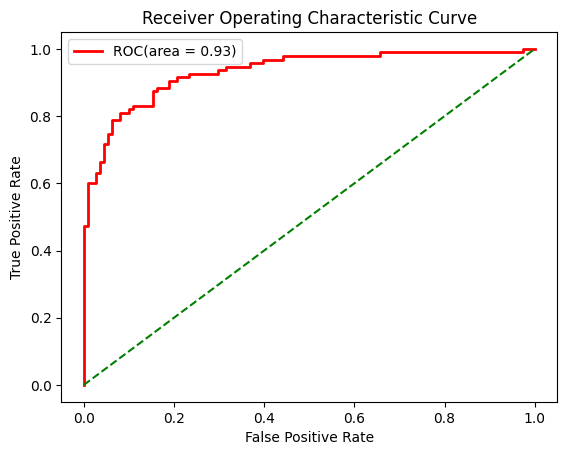

Supplement: Supplementary file 1 [file bioengineering-11-00399-s001.zip › Supplement 3/Model 2a 2b LARC case result/Model 2a Test 1 set 137 cases/Soft vote LARC AUC Confu. Matrix/Model 2a ROC T1 SoftVote 0.25.png]

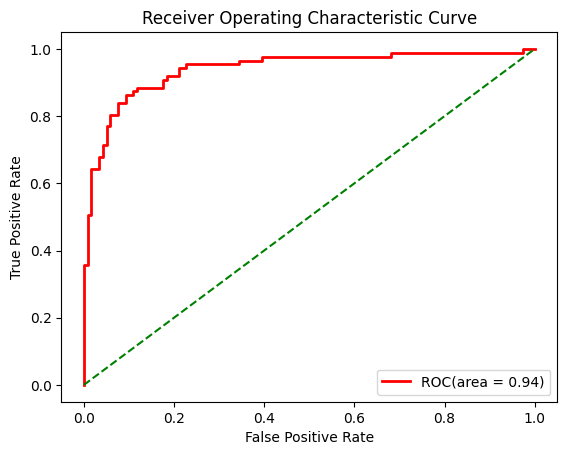

Supplement: Supplementary file 1 [file bioengineering-11-00399-s001.zip › Supplement 3/Model 2a 2b LARC case result/Model 2a Test 1 set 137 cases/Soft vote LARC AUC Confu. Matrix/Model 2a ROC T1 SoftVote 0.33.png]

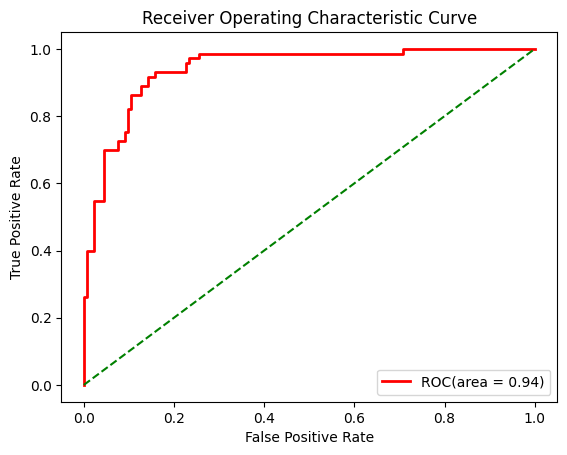

Supplement: Supplementary file 1 [file bioengineering-11-00399-s001.zip › Supplement 3/Model 2a 2b LARC case result/Model 2a Test 1 set 137 cases/Soft vote LARC AUC Confu. Matrix/Model 2a ROC T1 SoftVote 0.50.png]

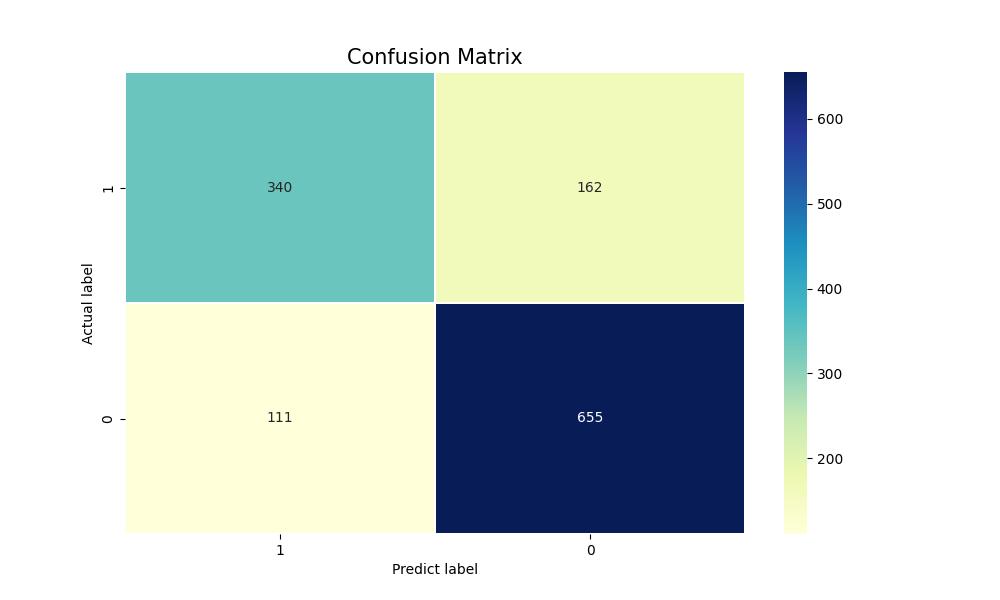

Supplement: Supplementary file 1 [file bioengineering-11-00399-s001.zip › Supplement 3/Model 2a 2b LARC case result/Model 2b Test 2 set 197 cases/CRM positive Images AUC Confu. matrix/Model 2b Confusion Matrix Test 2 Image.jpg]

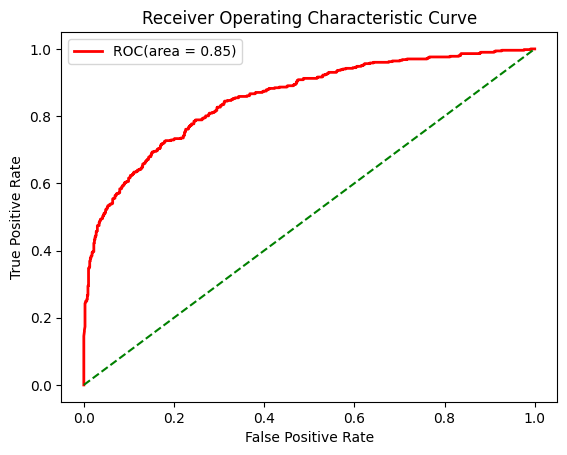

Supplement: Supplementary file 1 [file bioengineering-11-00399-s001.zip › Supplement 3/Model 2a 2b LARC case result/Model 2b Test 2 set 197 cases/CRM positive Images AUC Confu. matrix/Model 2b ROC Test 2 Image.png]

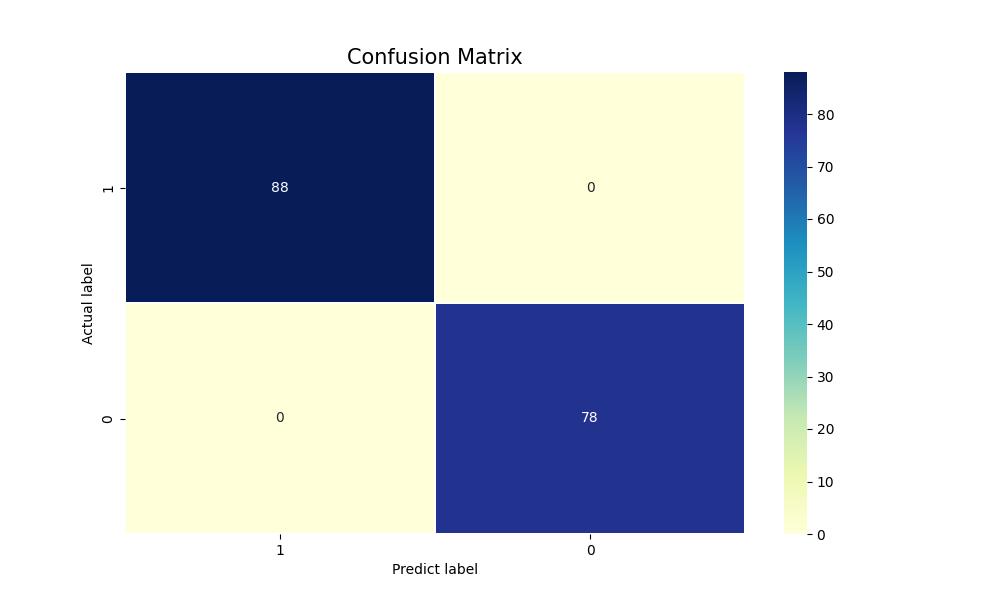

Supplement: Supplementary file 1 [file bioengineering-11-00399-s001.zip › Supplement 3/Model 2a 2b LARC case result/Model 2b Test 2 set 197 cases/Hard vote LARC AUC Confu. Matrix/Model 2b Confusion Matrix T2 HardVote +any one.jpg]

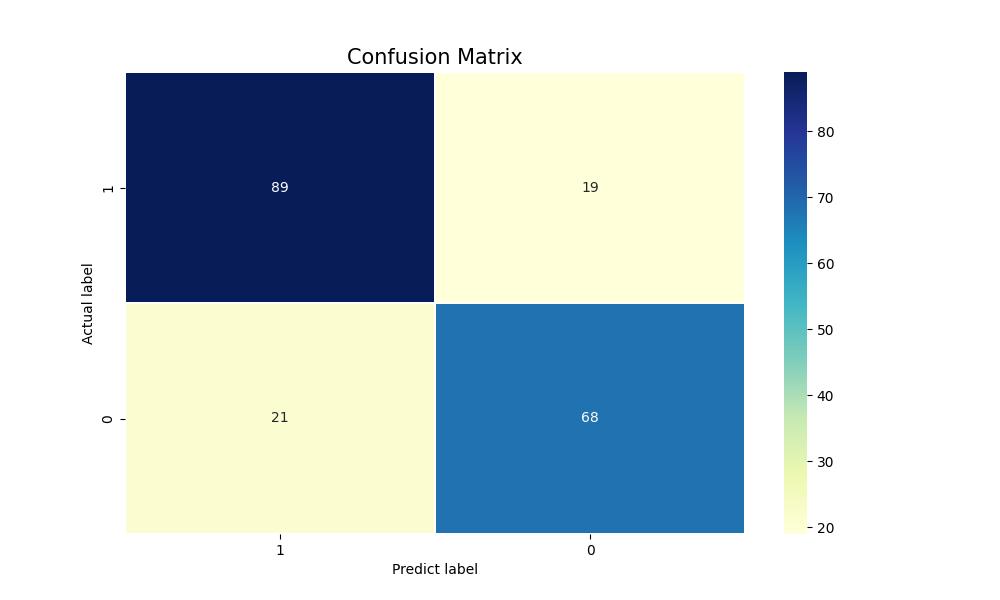

Supplement: Supplementary file 1 [file bioengineering-11-00399-s001.zip › Supplement 3/Model 2a 2b LARC case result/Model 2b Test 2 set 197 cases/Hard vote LARC AUC Confu. Matrix/Model 2b Confusion Matrix T2 HardVote 0.20.jpg]

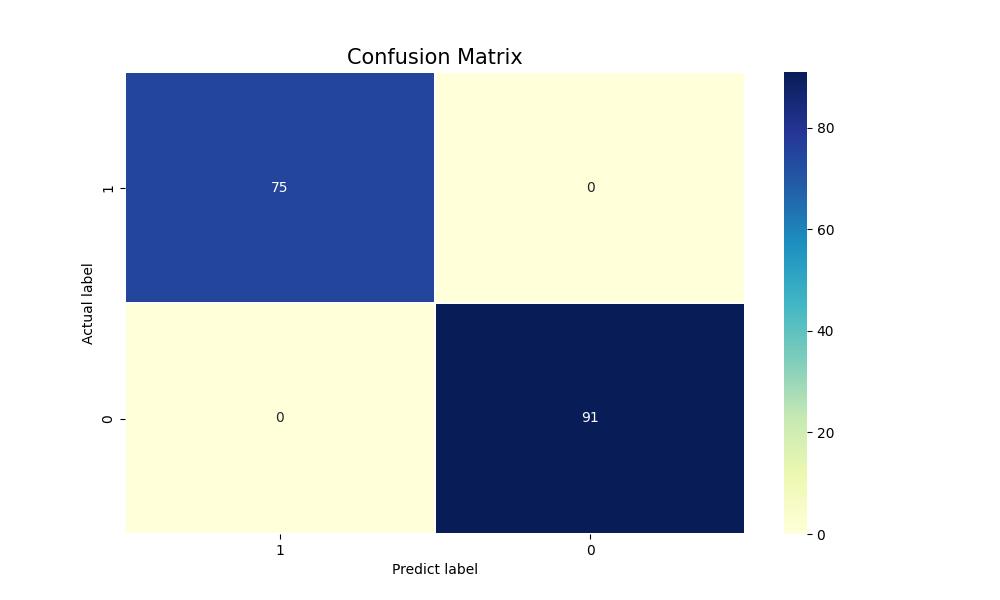

Supplement: Supplementary file 1 [file bioengineering-11-00399-s001.zip › Supplement 3/Model 2a 2b LARC case result/Model 2b Test 2 set 197 cases/Hard vote LARC AUC Confu. Matrix/Model 2b Confusion Matrix T2 HardVote 0.25.jpg]

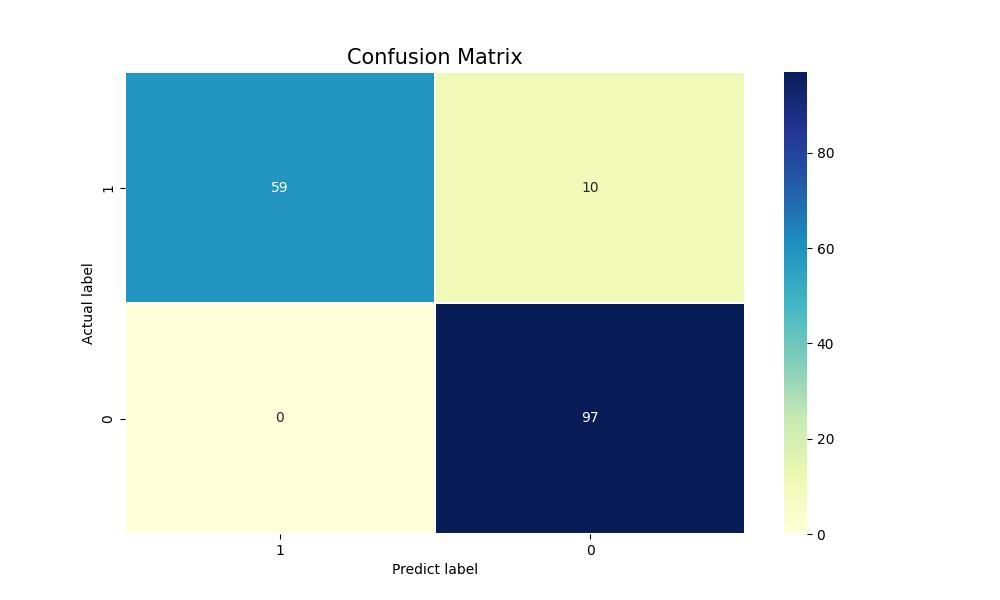

Supplement: Supplementary file 1 [file bioengineering-11-00399-s001.zip › Supplement 3/Model 2a 2b LARC case result/Model 2b Test 2 set 197 cases/Hard vote LARC AUC Confu. Matrix/Model 2b Confusion Matrix T2 HardVote 0.33.jpg]

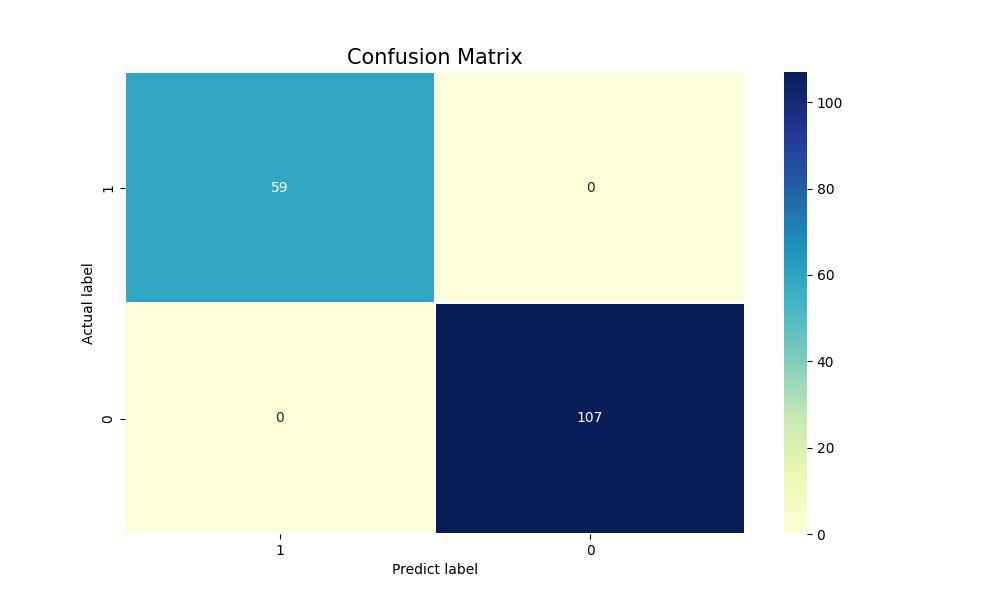

Supplement: Supplementary file 1 [file bioengineering-11-00399-s001.zip › Supplement 3/Model 2a 2b LARC case result/Model 2b Test 2 set 197 cases/Hard vote LARC AUC Confu. Matrix/Model 2b Confusion Matrix T2 HardVote 0.50.jpg]

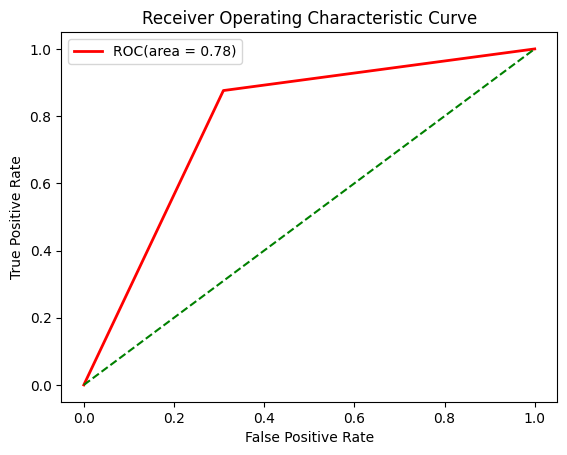

Supplement: Supplementary file 1 [file bioengineering-11-00399-s001.zip › Supplement 3/Model 2a 2b LARC case result/Model 2b Test 2 set 197 cases/Hard vote LARC AUC Confu. Matrix/Model 2b ROC T2 HardVote +any one.png]

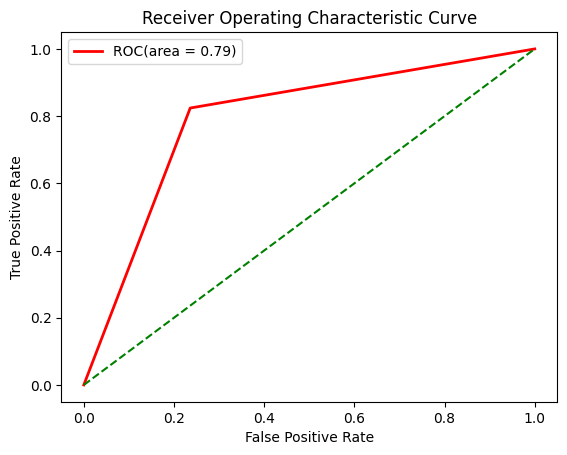

Supplement: Supplementary file 1 [file bioengineering-11-00399-s001.zip › Supplement 3/Model 2a 2b LARC case result/Model 2b Test 2 set 197 cases/Hard vote LARC AUC Confu. Matrix/Model 2b ROC T2 HardVote 0.20.png]

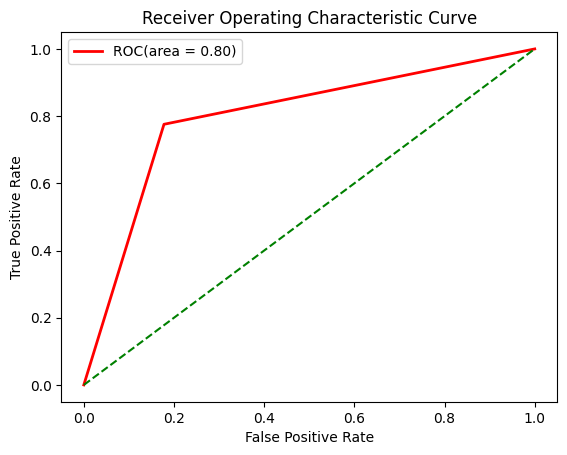

Supplement: Supplementary file 1 [file bioengineering-11-00399-s001.zip › Supplement 3/Model 2a 2b LARC case result/Model 2b Test 2 set 197 cases/Hard vote LARC AUC Confu. Matrix/Model 2b ROC T2 HardVote 0.25.png]

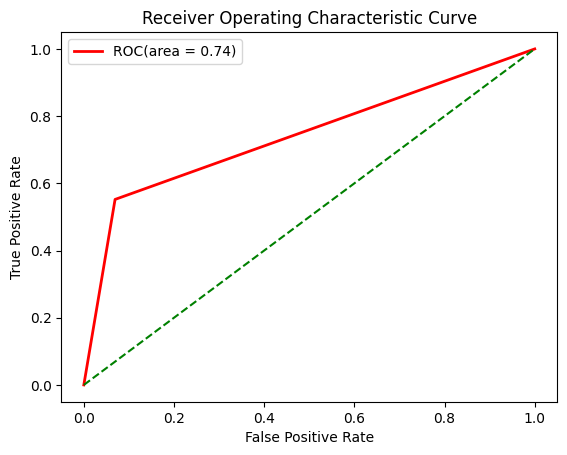

Supplement: Supplementary file 1 [file bioengineering-11-00399-s001.zip › Supplement 3/Model 2a 2b LARC case result/Model 2b Test 2 set 197 cases/Hard vote LARC AUC Confu. Matrix/Model 2b ROC T2 HardVote 0.33.png]

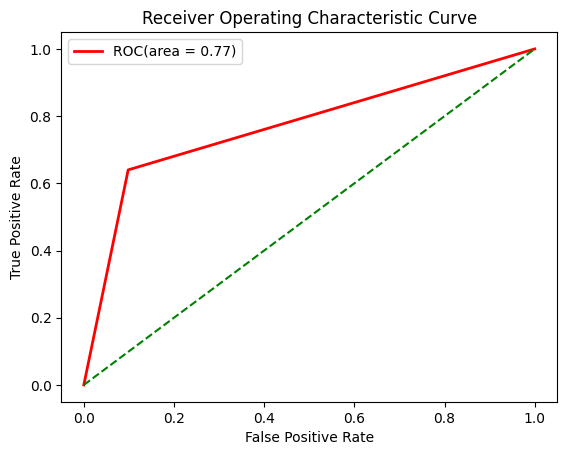

Supplement: Supplementary file 1 [file bioengineering-11-00399-s001.zip › Supplement 3/Model 2a 2b LARC case result/Model 2b Test 2 set 197 cases/Hard vote LARC AUC Confu. Matrix/Model 2b ROC T2 HardVote 0.50.png]

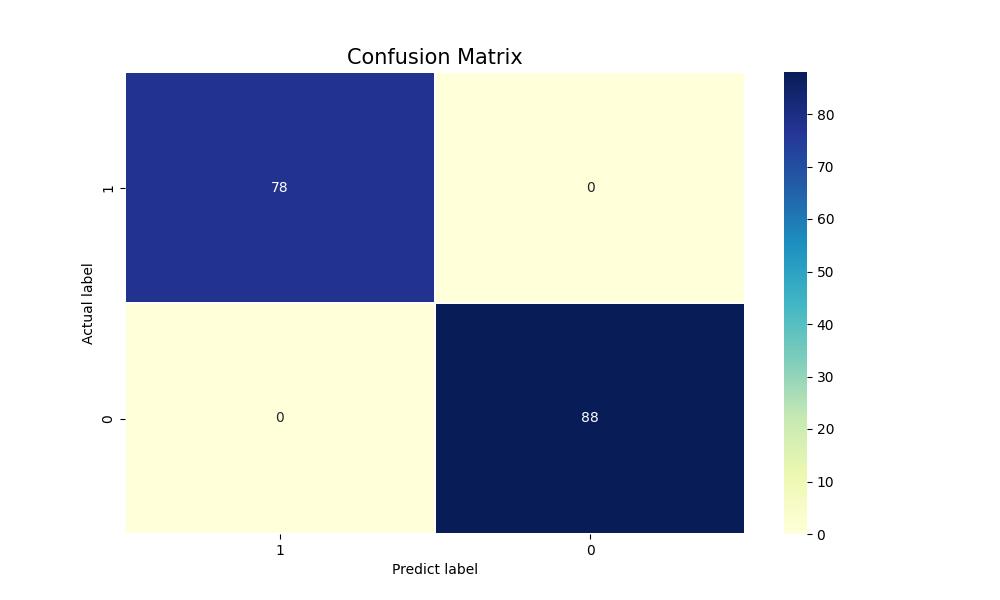

Supplement: Supplementary file 1 [file bioengineering-11-00399-s001.zip › Supplement 3/Model 2a 2b LARC case result/Model 2b Test 2 set 197 cases/Soft vote LARC AUC Confu. Matrix/Model 2b Confusion Matrix T2 SoftVote 0.20.jpg]

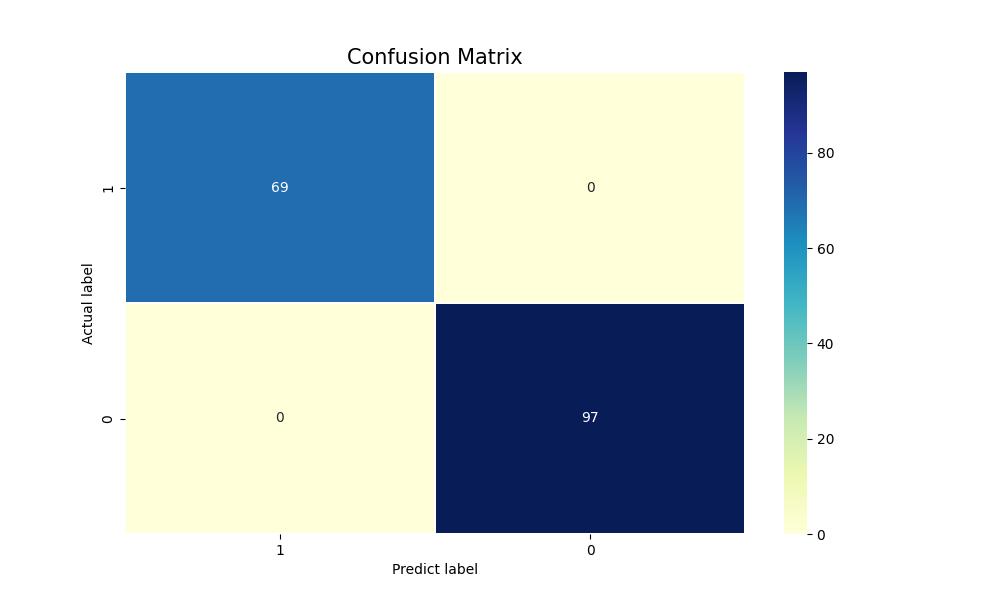

Supplement: Supplementary file 1 [file bioengineering-11-00399-s001.zip › Supplement 3/Model 2a 2b LARC case result/Model 2b Test 2 set 197 cases/Soft vote LARC AUC Confu. Matrix/Model 2b Confusion Matrix T2 SoftVote 0.33.jpg]

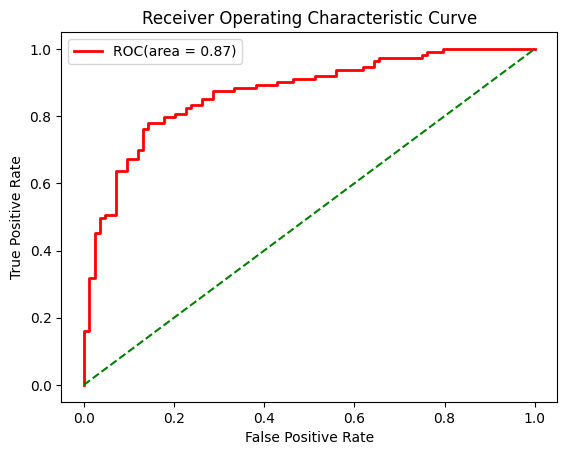

Supplement: Supplementary file 1 [file bioengineering-11-00399-s001.zip › Supplement 3/Model 2a 2b LARC case result/Model 2b Test 2 set 197 cases/Soft vote LARC AUC Confu. Matrix/Model 2b ROC T2 SoftVote +any one.png]

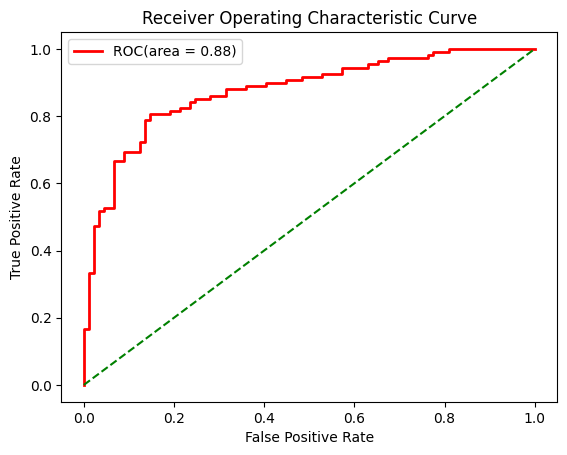

Supplement: Supplementary file 1 [file bioengineering-11-00399-s001.zip › Supplement 3/Model 2a 2b LARC case result/Model 2b Test 2 set 197 cases/Soft vote LARC AUC Confu. Matrix/Model 2b ROC T2 SoftVote 0.20.png]

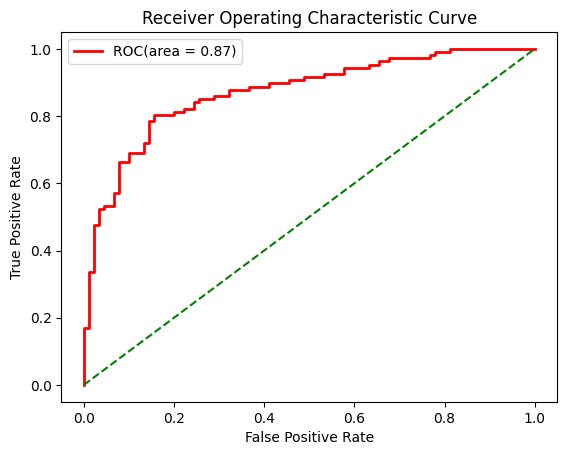

Supplement: Supplementary file 1 [file bioengineering-11-00399-s001.zip › Supplement 3/Model 2a 2b LARC case result/Model 2b Test 2 set 197 cases/Soft vote LARC AUC Confu. Matrix/Model 2b ROC T2 SoftVote 0.25.png]

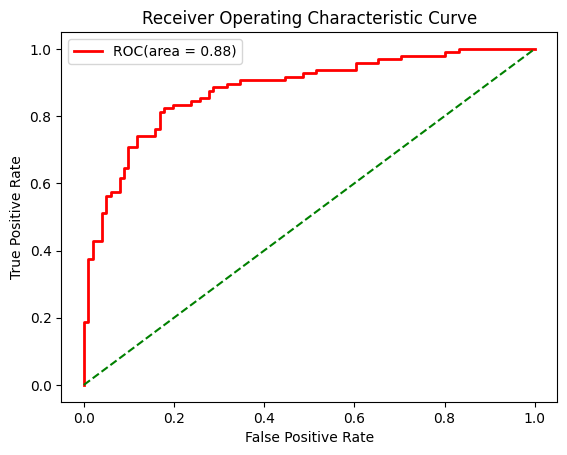

Supplement: Supplementary file 1 [file bioengineering-11-00399-s001.zip › Supplement 3/Model 2a 2b LARC case result/Model 2b Test 2 set 197 cases/Soft vote LARC AUC Confu. Matrix/Model 2b ROC T2 SoftVote 0.33.png]

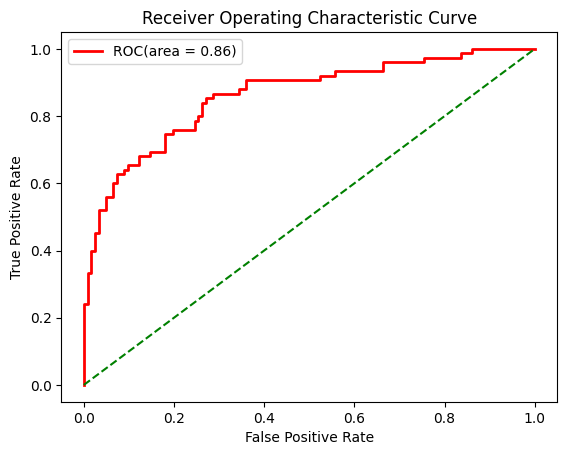

Supplement: Supplementary file 1 [file bioengineering-11-00399-s001.zip › Supplement 3/Model 2a 2b LARC case result/Model 2b Test 2 set 197 cases/Soft vote LARC AUC Confu. Matrix/Model 2b ROC T2 SoftVote 0.50.png]
